# Shedding Light on Primary Donors in Photosynthetic Reaction Centers

**DOI:** 10.3389/fmicb.2021.735666

**Published:** 2021-10-01

**Authors:** Michael Gorka, Amgalanbaatar Baldansuren, Amanda Malnati, Elijah Gruszecki, John H. Golbeck, K. V. Lakshmi

**Affiliations:** ^1^Department of Biochemistry and Molecular Biology, The Pennsylvania State University, University Park, PA, United States; ^2^Department of Chemistry and Chemical Biology and The Baruch ’60 Center for Biochemical Solar Energy Research, Rensselaer Polytechnic Institute, Troy, NY, United States; ^3^Department of Chemistry, The Pennsylvania State University, University Park, PA, United States

**Keywords:** primary donor, reaction center, heterodimer, homodimer, chlorophyll, electron paramagnetic resonance, density functional theory

## Abstract

Chlorophylls (Chl)s exist in a variety of flavors and are ubiquitous in both the energy and electron transfer processes of photosynthesis. The functions they perform often occur on the ultrafast (fs–ns) time scale and until recently, these have been difficult to measure in real time. Further, the complexity of the binding pockets and the resulting protein-matrix effects that alter the respective electronic properties have rendered theoretical modeling of these states difficult. Recent advances in experimental methodology, computational modeling, and emergence of new reaction center (RC) structures have renewed interest in these processes and allowed researchers to elucidate previously ambiguous functions of Chls and related pheophytins. This is complemented by a wealth of experimental data obtained from decades of prior research. Studying the electronic properties of Chl molecules has advanced our understanding of both the nature of the primary charge separation and subsequent electron transfer processes of RCs. In this review, we examine the structures of primary electron donors in Type I and Type II RCs in relation to the vast body of spectroscopic research that has been performed on them to date. Further, we present density functional theory calculations on each oxidized primary donor to study both their electronic properties and our ability to model experimental spectroscopic data. This allows us to directly compare the electronic properties of hetero- and homodimeric RCs.

## Introduction

Photosynthesis is perhaps one of the most important processes in nature. The ability of oxygenic photosynthesis to utilize the virtually inexhaustible supply of solar energy has powered the planet for billions of years. Some of the earliest signs of the presence of anoxygenic phototrophs, found in South Africa, date to more than 3.4 Ga (Tice and Lowe, 2004) and are believed to have used hydrogen (H_2_) and/or iron (Fe) as the source reducing equivalents for carbon fixation (Widdel et al., 1993; Fischer et al., 2016). In the modern era, evolutionary history is marked by the advent of cyanobacteria [∼ 2.4 Ga (Hofmann, 1976; Bekker et al., 2004; Rasmussen et al., 2008)] that utilizes the free energy provided by the sun to generate a highly oxidizing species that splits water, producing reducing equivalents that are ultimately stored as NADPH or ‘biohydrogen’ for use in atmospheric CO_2_ fixation. This process has evolved to be incredibly efficient, with a quantum efficiency of 0.97 for energy capture (Şener et al., 2011). Furthermore, the energy loss in these systems is mitigated by electron-transfer processes where approx. 60% of the energy of a red photon is conserved (Ke et al., 1973).

**FIGURE 1:**
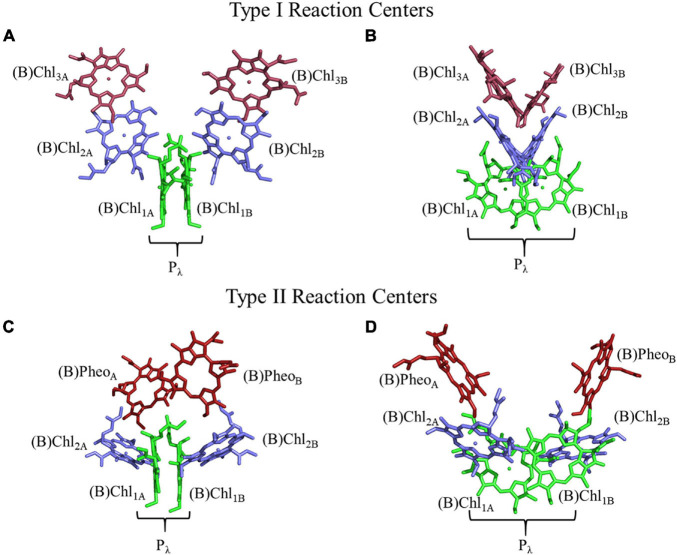
Structure and orientation of early (B)Chl acceptors in Type I (top) and Type II (bottom) RCs viewed from different angles. **(A,C)** A parallel orientation of the primary donor macrocycle planes, and **(B,D)** a perpendicular orientation of the primary donor macrocycle planes. The (B)Chl molecules of the primary donor, (B)Chl_1_, are shown in green, (B)Chl_2_ in blue, and (B)Chl_3_/(B)Pheo in red. The two (pseudo-)symmetric branches of cofactors are denoted as ‘A’ and ‘B,’ respectively.

Light-driven electron-transfer in photosynthesis originates in a structure known as the reaction center (RC). Reaction centers are large, multi-subunit pigment-protein complexes that harvest light energy through a network of internal or external chlorophyll (Chl) or bacteriochlorophyll (BChl) molecules and store the energy through charge separation and mobilization (Golbeck, 2006; Vinyard et al., 2013). Photoexcitation of the RC leads to rapid charge separation between two (or more) (B)Chl molecules bound in the polypeptide core of the RC. The initial charge separation is subsequently stabilized by electron transfer through a series of cofactors, which extends the lifetime of charge separation from the picosecond to the physiologically relevant millisecond time scale. There are two types of RCs in nature, Type I and Type II, that can be differentiated in several ways. The most obvious difference is the identity of the terminal electron acceptors of the RCs. The electron transfer pathway(s) in Type I RCs utilize iron-sulfur ([4Fe–4S]) clusters, whereas, Type II RCs use quinone molecules as terminal acceptors. Moreover, the identity and orientation of the primary electron acceptor(s) are different in Type I and II RCs (Figure 1). The primary acceptor of Type II RCs is a (bacterio)pheophytin [(B)Pheo], lacking a central metal ion, whereas the primary donor of the RC [with the exception of the RC from *Acaryochloris marina* (Chen et al., 2005; Tomo et al., 2007)] is a (B)Pheo derivative, (B)Chl, of the same type. In contrast, Type I RCs always contain (B)Chl *a* (or related derivative) as the primary acceptor regardless of the identity of the primary donor (Orf et al., 2018).

**FIGURE 2:**
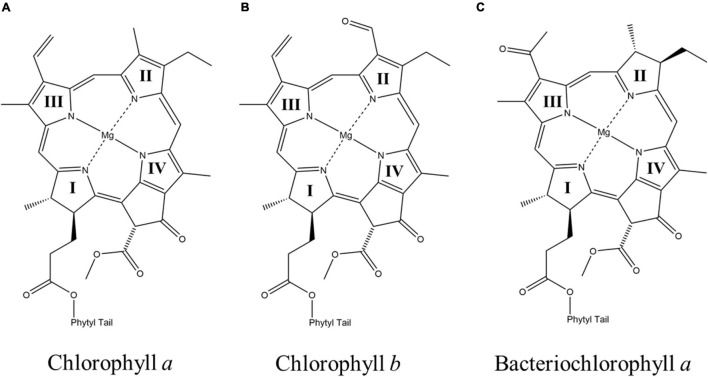
The chemical structures of **(A)** chlorophyll *a* (Chl *a*) **(B)** chlorophyll *b* (Chl *b*), and **(C)** bacteriochlorophyll *a* (BChl *a*). The nitrogen atoms of the four pyrrole rings in each macrocycle are labeled as I, II, III, and IV, respectively.

Reaction centers can be further categorized as homodimers and heterodimers. The core of homodimeric RCs is comprised of a dimer of polypeptide subunits that are encoded by a single gene, *e.g.*, the RC of *Heliobacterium modesticaldum*. Homodimeric RCs tend to be simpler in function, containing fewer polypeptide subunits and electron-transfer cofactors (Gisriel et al., 2017). While there are two branches of symmetric cofactors available for electron transfer in homodimeric RCs, it is predicted that since there is no means of differentiating between the two branches, it is axiomatic that both are employed equally. In contrast, heterodimeric RCs contain two distinct polypeptide subunits with reasonably high sequence homology. These RCs also contain two branches of cofactors for electron transfer, which appear symmetric. However, the heterogeneity of the protein-matrix leads to significant differences in the usage and time scales of electron transfer. For example, in the heterodimeric Type I RC of cyanobacteria, photosystem I (PS I), electron transfer in the A-branch is preferred over the B-branch by a factor of ∼ 2 (Agalarov and Brettel, 2003; Poluektov et al., 2005). This situation is pushed to the extreme in heterodimeric Type II RCs, such as, photosystem II (PS II) and the bacterial RC (bRC) from *Rhodobacter (Rba.) sphaeroides* and *Rhodopseudomonas (Rps.) viridis*, where the A-branch is exclusively used for electron transfer (Mimuro et al., 1995; Wakeham and Jones, 2005; Williams and Allen, 2009; Vinyard et al., 2013). Although the protein matrix effects responsible for the free energy differences that lead to branch specificity in RCs are yet to be fully elucidated (Wakeham and Jones, 2005), the study of site-specific genetic variants has yielded partial insight on the factors that influence directionality in heterodimeric RCs (Kirmaier et al., 1991, 1999; de Boer et al., 2002).

(Bacterio)chlorophyll molecules are amongst the most ubiquitous cofactors in RCs, with BChls commonly found in anoxygenic phototrophic bacteria, such as, the green sulfur bacterium, *Chlorobaculum tepidum*, and purple bacterium, *Rba. sphaeroides*, while Chls are found in oxygenic phototrophs, such as, higher plants and cyanobacteria (Oren, 2011). Both Chl and BChl molecules are tetrapyrrole macrocycles that bind a central metal ion, which is usually Mg^2+^ (but also Zn^2+^ in rare cases) (Tomi et al., 2007; He et al., 2019; Charles et al., 2020). The (B)Chl molecules can be distinguished by the extent of delocalization of the π-system across the tetrapyrrole macrocycle, which leads to a shift in the absorbance maximum, with the BChls absorbing farther to the red (Strain and Svec, 1966). The (B)Chls can be further divided into different ‘flavors,’ differentiated by the substituents on the macrocycle and are often denoted by a letter, such as, Chl *a* and Chl *b*. There are six Chl molecules, Chl *a*, 8^1^-hydroxy-Chl *a*, divinyl-Chl *a/b*, Chl *b*, Chl *d*, and Chl *f*, and six BChl molecules, BChl *a*, BChl *b*, BChl *c*, BChl *d*, BChl *e* and BChl *g*, currently known (Chew and Bryant, 2007). Divinyl (DV)Chls are a variation of Chls, where the ethyl group of the pyrrole ring (II) is changed to a vinyl group (Steglich et al., 2003; Ito and Tanaka, 2011; Barrera-Rojas et al., 2018). This relatively minor change allows for absorption of blue light with virtually no impact on the lifetime of the excited state (see below) or their fluorescence quantum yield (Steglich et al., 2003). They are predominately found in *Prochlorococcus*, a deep-sea growing marine cyanobacterium that uses DVChls to absorb the plentiful blue light found deeper in the water column (Ralf and Repeta, 1992; Moore et al., 1995; Partensky et al., 1999). In general, the various substituents on the (B)Chl macrocycles lead to changes of the extended π-system, which results in shifts of the absorbance bands. The structures of three of the most common (B)Chl molecules are shown in Figure 2. In addition to a functionalized tetrapyrrole macrocycle, (B)Chls contain a long hydrophobic tail that is often a phytol group (Chew and Bryant, 2007), but can also be other substituents, such as, farnesol in heliobacteria (Hb) (Kobayashi et al., 1991) and green sulfur bacteria (Gsb) (Frigaard et al., 2006). While the function of the phytol tail is likely to serve as a lipophilic anchor within the hydrophobic trans-membrane region(s) of proteins, it has been suggested that steric effects can impact the electronic properties of the macrocycle, as well as control the chelation properties of the central metal ion (Fiedor et al., 2008).

The arrangement of (B)Chl molecules in Type I and Type II RCs are presented in Figure 1. For the purposes of this review article, we will follow a common nomenclature for the (B)Chl cofactors across a variety of hetero- and homodimeric RCs. The two branches of cofactors will be denoted as ‘A’ and ‘B,’ as is common for Type I RCs. It should be noted that for Type II RCs, ‘A’ corresponds to the ‘L’ and ‘D1’ branches of the bRC and PS II, respectively. Similarly, ‘B’ refers to the ‘M’ and ‘D2’ branches of the bRC and PS II, respectively. The two closely spaced (B)Chl molecules near the luminal side of each RC are termed Chl_1__X_, where X is the associated A- or B-branch (Figure 1). These dimeric (B)Chl_1__A/__1__B_ molecules comprise the primary donor, P_λ_, where λ is the wavelength of maximal absorbance. As both the chemical identity of the pair of (B)Chls in the primary donor, and the surrounding electrostatic environment vary between species, the value of λ is unique for each RC. We term the second pair of (B)Chl molecules of the RC as ‘(B)Chl_2A/2B_.’ The (B)Chl in this position is also commonly referred to as the ‘accessory (B)Chl,’ as well as B_A/B_ and Chl_A/B_ in the bRC and PS II, respectively. The orientation of (B)Chl_2A/2B_ in Type I RCs is nearly perpendicular to the membrane plane, and parallel to the plane of (B)Chl_3A/3B_, while Type II RCs display a roughly parallel orientation of (B)Chl_2A/2B_ with the membrane plane and are almost perpendicular to the neighboring (B)Pheo. We label the final pair of (B)Chl molecules in the RC as (B)Chl_3A/3B_, but we refer to these cofactors as (B)Pheo_A/B_ in Type II RCs (this in keeping with the nomenclature used for Type II RCs). The (B)Chl_3A/3B_ molecules are also commonly referred to as H_A/B_ and A_0_ in the bRC and PS I, respectively.

There are several reasons that (B)Chl molecules are ubiquitous in photosynthesis [reviewed in Mauzerall et al. (1976) and Björn et al. (2009)], and in the interest of brevity we will only address a few reasons here. First, Chl molecules have an extended and modifiable π-system, drastically reducing the energy gap between the highest occupied molecular orbital (HOMO) and lowest unoccupied molecular orbital (LUMO), which allows for absorption of photons in the visible region of the electromagnetic spectrum. As described below, slight modification of the π-system of Chl derivatives allows for the absorption of different wavelengths of light. This, in turn, allows organisms to exist in a variety of different ecological niches, or to switch their relative Chl abundance in response to changes in the environment (Humbeck et al., 1984). Second, the extended lifetime of Chl excitation allows for efficient photochemistry. It should be noted that the time scale of Chl de-excitation *in vivo* is ∼ 0.3–2.3 ns (Brody and Rabinowitch, 1957; Mar et al., 1972; Morales et al., 2001) which is marginally faster than *in vitro* [5.1 and 3.9 ns for Chl *a* and Chl *b*, respectively (Brody and Rabinowitch, 1957; Brody, 2002)]. The enhanced time scale of de-excitation of Chl molecules *in vivo* is due to the interaction with the surrounding protein matrix. Regardless, these values are significantly slower than charge separation events, which occur with lifetimes of ∼ 100 fs (Shelaev et al., 2010) therefore providing ample time for photochemistry to occur.

**FIGURE 3:**
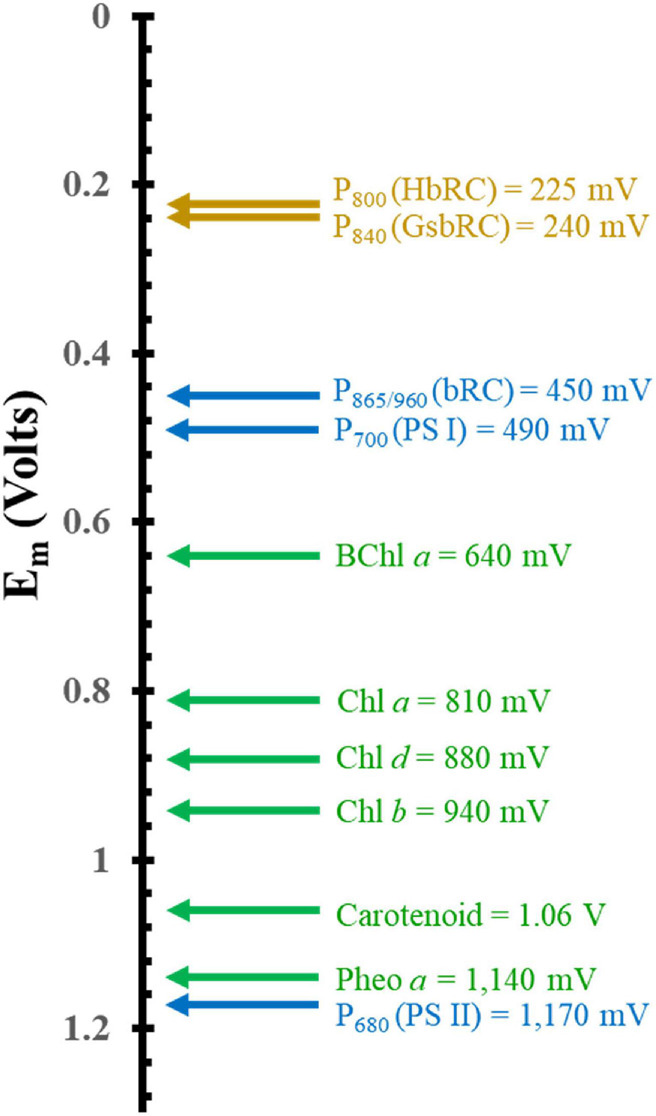
Comparison of the mid-point potential of a variety of (B)Chls *in vitro* and primary donors *in vivo*. Isolated *in vitro* values are depicted in green and hetero and homodimeric primary donors are in blue and dark yellow, respectively.

Another important factor that dictates the use of (B)Chls is the tunability of the electronic properties through interactions with the surrounding protein matrix. Figure 3 showcases the redox potentials (both *in vitro* and *in vivo*) for a variety of monomeric (B)Chl molecules and primary donors of RCs. While smart protein-matrix interactions are not unique to (B)Chls, there are a multitude of methods by which the protein matrix can affect the redox properties of a Chl monomer or multimer (Srinivasan and Golbeck, 2009; Olson et al., 2013; Allen and Williams, 2014). One method for shifting a (B)Chl toward either a more positive or more negative redox potential is through the addition or elimination of charged residues within a distance of ∼10 Å of the cofactor (Williams et al., 2001; Johnson and Parson, 2002; Johnson et al., 2002; Ishikita et al., 2006). This method has immense flexibility in altering the potential depending on the charge of the added residue, with the impact being roughly controlled by the distance to that residue. The amplitude of this shift has been observed to vary for different cofactors, with a ∼50–60 mV shift (in either direction) for P_865_ (Allen and Williams, 2014) from the bRC, and up to +144 mV for the phylloquinones and iron-sulfur cluster, F_X_, of PS I (Karyagina et al., 2007; Srinivasan and Golbeck, 2009). Hydrogen bonding provides yet another means of tuning the redox properties of cofactors, with the added benefit of serving as a structural component. The redox potential of P_865_ has been shown to increase by 60–125 mV when a hydrogen bond is added, while removal of a preexisting bond decreases the potential by a similar amount (Allen and Williams, 2014). This effect is mirrored in the quinone acceptors of PS I (Srinivasan and Golbeck, 2009) and the bRC (Taguchi et al., 2013; Vermaas et al., 2015), to such an extent that the Gly225_L_ residue that is hydrogen bonded to the secondary quinone acceptor, Q_B_, of the bRC is thought to be primarily responsible for the difference of the redox potential between the primary and secondary quinone, Q_A_, and Q_B_ (Wraight, 1979; Kleinfeld et al., 1984a; Taguchi et al., 2013). Indeed, the addition of a hydrogen bond to the phylloquinone acceptor, A_1_, in PS I (by altering the axial ligand of the Chl_3_ molecule) shifts its redox potential such that it is too positive to participate in forward electron transfer (Sun et al., 2014; Gorka et al., 2021a).

While the addition of charged residues and hydrogen bonds constitute common strategies for controlling the redox properties of cofactors in proteins, there are aspects that are unique to (B)Chls in RCs. First, a typical strategy involves π-stacking of a nearby amino acid residue or neighboring cofactor. This has an ancillary benefit of facilitating the binding of cofactors (Mao et al., 2003), however, it is limited in that π-stacking only alters the potential in one direction, *i.e.*, toward a more negative potential. This strategy has been used to alter the potential of the phylloquinone cofactors, A_1A/1B_, of PS I leading to a decrease in the potential from approx. 27 mV (Srinivasan and Golbeck, 2009) to 150 mV (Kaupp, 2002) by the presence of a π-stacked Trp679_A_/Trp677_B_ residue. To date, there have been few studies of this phenomenon in (B)Chl molecules, but given the extended π-system of the (B)Chls, and the recent discovery of a π-stacked Phe residue in the green-sulfur bacterial (Gsb)RC (Chen et al., 2020), this appears to be a feasible approach. Additionally, the wide variety of available axial ligands that are employed in the binding of (B)Chls in RCs can have a sizeable impact on the redox potential (Heimdal et al., 2007). While most Chl molecules in the photosynthetic antenna complexes are bound by the imidazole of a His residue, as expected by hard-soft acid-base theory (Pearson, 1963), other groups are frequently employed as axial ligands as well. For example, a Met residue is used to bind the Chl molecules, Chl_3A/3B_, involved in electron transfer in PS I (Jordan et al., 2001), and changes to this axial ligand have been demonstrated to significantly impact the electronic (Gorka et al., 2021a) and electron-transfer (Cohen et al., 2004; Dashdorj et al., 2005; van der Est et al., 2009; Santabarbara et al., 2010) properties of Chl_3A/3B_. Finally, planar ring distortions can lead to changes in the absorptive features of (B)Chl molecules (Zucchelli et al., 2007). These protein matrix effects work in tandem to help tune the spectral, electronic, and redox properties of (B)Chl molecules so as to serve multiple functions, even within the same RC.

(B)Chls are critical to photosynthesis as they are not only used to harvest solar energy but these are the only pigments used to generate a charge-separated state, and play an integral role in photosynthetic electron transfer. Light harvesting in photosynthesis is often performed through a pool of densely packed (B)Chl molecules that allow energy transfer to neighboring pigments over very short time scales using fluorescence resonance energy transfer (FRET) (Oppenheimer and Schwinger, 1941; Şener et al., 2011). In the case of PS I, the antenna pool is contained within the RC itself (Jordan et al., 2001; Şener et al., 2011) and is comprised of nearly 100 Chl molecules, although additional antenna complexes are also formed under differing physiological conditions (Bibby et al., 2001). For example, the bacterial reaction center (bRC) and PS II contain light-harvesting complexes (LHCs), termed LHC-I and LHC-II, which aid in light harvesting and energy transfer through closely packed (B)Chl molecules (Deisenhofer et al., 1985; McDermott et al., 1995; Koepke et al., 1996; Papiz et al., 2003; Roszak et al., 2003; Şener et al., 2011; van Amerongen and Croce, 2013). This process can also occur externally through energy transfer from the chlorosome to the RC via the Fenna-Matthews-Olson (FMO) protein (Olson, 2004), as is the case for green sulfur bacteria (Olson, 2013). However, energy transfer to the RC in green sulfur bacteria is unusually inefficient (Blankenship et al., 1995; Francke et al., 1996; Neerken et al., 1998; Oh-oka et al., 1998; Olson, 1998), with an estimated value of only ∼ 30%. The energy of an absorbed photon migrates across this network of (B)Chl molecules until the energy finds its way to a thermodynamic well in the RC, known as the trap. Excitation of the trap leads to the charge-separation reaction in the RC. This process occurs on ultrafast time scales, from fs (Shelaev et al., 2010; Song et al., 2021) to ps (Holzapfel et al., 1989; Peloquin et al., 1994; Holzwarth and Müller, 1996), and has thus been difficult to study. Recent advances in theoretical and experimental techniques have led to several proposed models for charge separation in RCs. While a detailed analysis of every model is outside the scope of this review article, we will provide brief descriptions of the types that have been put forth. It should also be noted that given the variations in cofactor identity, structure, and organization in the different RCs, the exact mechanism of charge separation may vary between them. Therefore, the models for each RC are described in greater detail in the respective sections [for a comprehensive review, please see Savikhin and Jankowiak (2014)].

**SCHEME 1:**
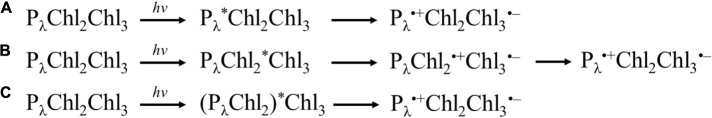
Simple archetypes of charge separation models. **(A)** Excitation of primary donor, P_λ_, leading to charge separation between P_λ_ and Chl_3_. **(B)** Excitation of Chl_2_, leading to charge separation between Chl_2_ and Chl_3_, followed by the hole on Chl_2_ being filled by P_λ_. **(C)** Excitation of highly coupled P_λ_, Chl_2_ (and potentially Chl_3_). Please note that Chl_3_ is a Pheo in Type II RCs.

In general, three categories of models for charge-separation have been proposed in the literature, where the process originates either at the: (i) primary donor, (ii) accessory Chl, or (ii) a larger group of highly coupled cofactors. Shown in Scheme 1 is a simple representation of the models.

For many years, the primary donor (P) had been thought of as the initial site for charge separation (White et al., 1996; Savikhin et al., 2000; Gibasiewicz et al., 2001; Gobets and van Grondelle, 2001; Gobets et al., 2001; Melkozernov, 2001; Savikhin and Jankowiak, 2014), whereby this electronically coupled special pair of (B)Chl molecules serves as an energy sink for the antenna complexes. While it was unclear whether the act of charge separation or energy transfer to the trap was the limiting factor, all of the models assumed that the low-potential reductant emerged from the primary donor, initially reducing (B)Chl_2_. Advances in ultrafast optical spectroscopy have made it possible to investigate the femto- and picosecond transient states of the RCs, which led to various proposed models for the charge separation reaction. A thorough review of ultrafast optical spectroscopy methods can be found in Berera et al. (2009) and Schlau-Cohen et al. (2012). Ultrafast spectroscopy has been employed for the study of the bRC (Van Brederode et al., 1997; van Brederode et al., 1998, 1999; van Stokkum et al., 1997; Vos et al., 1997; van Brederode and van Grondelle, 1999; Konar et al., 2018; Ma et al., 2019), PS II (Schatz et al., 1987; Peterman et al., 1998; Novoderezhkin et al., 2007; Myers et al., 2010), PS I (Müller et al., 2003, 2010; Holzwarth et al., 2006a; Cherepanov et al., 2017, 2020, 2021), and the HbRC (Kojima et al., 2020; Song et al., 2021). Early research on the bRC demonstrated that charge separation with the successful formation of the reduced Q_A_^⋅–^ state still occurs in site-directed genetic variants with significantly slower energy transfer from BChl_2A_^∗^ to P_865_ (Van Brederode et al., 1997; van Brederode et al., 1998). These results, coupled with the observation that direct excitation of BChl_2A_ resulted in the formation of P_865_^∗^, led to a model in which BChl_2A_ was the genesis of the charge-separated state. Other studies around the same time period suggested instead that the early BChls should be considered a hexamer, where both P_865_ and BChl_2_ can be coherently excited (Vos et al., 1997). Much later, multiple two-dimensional electronic spectroscopy with better time resolution showed that when vibrational and electronic factors are considered, it is more reasonable to conclude that P_865_ initiates charge separation (Niedringhaus et al., 2018; Ma et al., 2019) and that P_865_ and BChl_2A/2B_ are electronically coupled, allowing for excited state migration (Konar et al., 2018). In principle, this could lead to the presence of multiple redundant mechanisms of charge separation in the system.

Analogous models for charge separation were also proposed for PS I using ultrafast spectroscopy methods. Ultrafast difference spectroscopy of wild-type (WT) PS I showed a mixture of the excited P_700_^∗^ state and the radical states associated with the charge-separated state, P_700_^⋅+^Chl_2A/B_^⋅–^. Moreover, perturbation of the protein environment surrounding the Chl_2A/2B_ cofactors through site-directed genetic variants appeared to slow the formation of the charge-separated state in the symmetric branch, e.g., changes to Chl_2B_ suppressed the formation of P_700_^⋅+^Chl_2A_^⋅–^ state. This suggested the presence of a highly coupled system of Chl molecules in PS I. Most recently, ultrafast spectroscopy of both WT PS I and genetic variants of the protein environment in the vicinity of the Chl_2_ cofactor have suggested the formation of an initial excited state (Chl_2A_P_700_Chl_2B_)^∗^ (Cherepanov et al., 2020, 2021), which rapidly leads to the formation of the first charge-separated state, P_700_^⋅+^Chl_2_^⋅–^.

In contrast, Müller and coworkers have proposed a mechanism of charge separation in PS I that originates at Chl_2_ (Müller et al., 2003; Holzwarth et al., 2006a), where the first charge-separated state is Chl_2_^⋅+^Chl_3_^⋅–^. Here, the hole on Chl_2_ was suggested to be reduced by P_700_ (Chl_1_), forming the first stable charge-separated state, P_700_^⋅+^Chl_3_^⋅–^. Theoretical studies of PS II have found that this is a promising model, as Chl_2_ was observed to have the lowest site energy of the initial Chl/Pheo molecules in the core (Sirohiwal et al., 2020). Recent findings have indicated common ground with both of the models described above, suggesting that the initial four (or six) Chl molecules of PS I are highly electronically coupled, and participate in charge separation to various extents. Similar models have also been proposed for the homodimeric RC from *H. modesticaldum*, invoking both the BChl_2_/BChl_3_ pair (Song et al., 2021) and/or all six core BChl molecules (Kojima et al., 2020; Song et al., 2021) in the excited state. It should be noted that these models are not necessarily mutually exclusive as different models likely take priority under different excitation conditions and may vary between RCs.

With the recent availability of the X-ray crystal and cryo-electron microscopy structures of the HbRC and GsbRC (Gisriel et al., 2017; Chen et al., 2020), respectively, we have the unique opportunity to compare the electronic structure of the primary donors in a variety of RCs. The goal of this article is to review research on the geometric and electronic structure of a variety of primary donors of hetero and homodimeric RCs, with an emphasis on pulsed electron paramagnetic resonance (EPR) and density functional theory (DFT) methods. We will focus on six RCs: (i) The heterodimers, P_700_ of Photosystem I (PS I), P_680_ of Photosystem II (PS II), P_865_ and P_960_ of the bRCs from *Rba. sphaeroides* and *Rps. viridis*, respectively, and (ii) homodimers, P_800_ of *H. modesticaldum* and P_840_ of the green-sulfur bacterium, *C. tepidum*.

## Geometric Structures

### Geometric Structure of the Primary Donor of Heterodimeric Reaction Centers

#### The Primary Donor, P_700_, of Photosystem I

**FIGURE 4:**
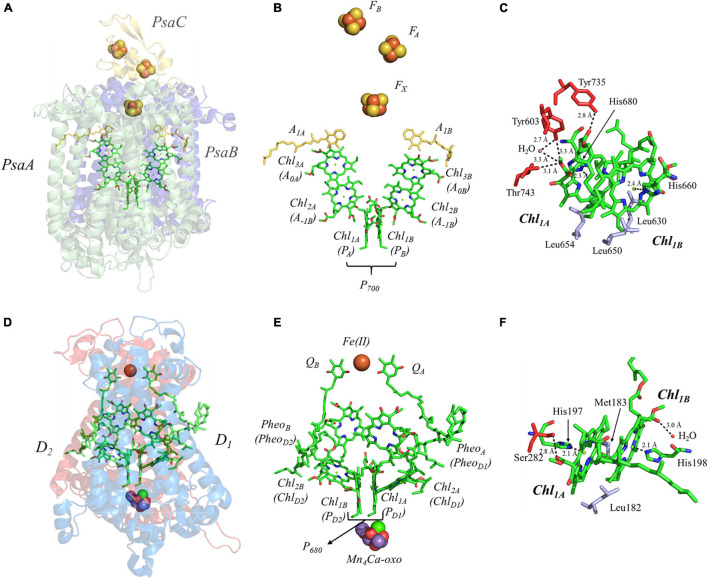
**(A)** The core subunits (PsaA, green; PsaB, blue; and PsaC, yellow) and cofactors of PS I as observed by X-ray crystallography (PDB ID: 1jb0), **(B)** the cofactors that participate in the primary electron transfer pathway of PS I and **(C)** the binding pocket of the primary donor, P_700_. **(D)** The core subunits (D1, red; D2, blue) and cofactors of PS II as observed by X-ray crystallography (PDB ID: 3wu2), **(E)** the cofactors that participate in the primary electron transfer pathway of PS II and **(F)** the binding pocket of the primary donor, P_680_. Commonly used labels are in parentheses. Part **(C,F)** emphasize the prominent protein-matrix effects of the primary donor of PS I and PS II, respectively, where the residues that are hydrogen bonded to the primary donors are shown in red, and nearby non-polar or π-stacked residues are colored in gray.

We begin by describing the extensively studied primary donor, P_700_, of PS I. Cyanobacterial PS I [reviewed in Golbeck (2006)] is a composed of 12 or 13 polypeptide subunits that bind the light-harvesting and electron-transfer cofactors. There are 90 Chl *a* molecules that function as the light-harvesting antenna while the electron-transfer cofactors are comprised of two pseudo-symmetric branches of six Chl *a* molecules (Chl_1A/1B_, Chl_2A/2B_, and Chl_3A/3B_), two phylloquinones (A_1A/1B_), and three [4Fe–4S] clusters, F_X_, F_A_ and F_B_ (Figures 4A,B). The electron-transfer cofactors are largely bound in the transmembrane PsaA and PsaB polypeptide subunits, with the terminal [4Fe–4S] clusters, F_A_ and F_B_, bound by PsaC on the stromal side of the RC (Figure 4A).

There is currently a lack of consensus on the mechanism of charge separation in PS I. Early models had suggested that P_700_ functioned as the source of the electron (White et al., 1996; Savikhin et al., 2000; Gibasiewicz et al., 2001; Gobets and van Grondelle, 2001; Gobets et al., 2001; Melkozernov, 2001; Savikhin and Jankowiak, 2014), whereby the dimerization of Chl_1A_/Chl_1B_ created a lower energy site that traps the excitation energy. Subsequently, it was proposed that Chl_2_, instead of acting as the first electron acceptor, served to trap the energy and was the source of the charge-separated state (Müller et al., 2003; Holzwarth et al., 2006a). It was proposed that Chl_2_ would reduce Chl_3_ and the hole that was generated would migrate to P_700_, ultimately creating the charge-separated P_700_^⋅+^Chl_3_^⋅–^ state. However, theoretical studies have suggested that the redox potentials of the Chl_2_/Chl_3_ pair make this mechanism thermodynamically unlikely (Ptushenko et al., 2008). More recently, advances in theoretical and experimental methods have led to a highly electronically coupled model, wherein (P_700_Chl_2A_Chl_2B_)^∗^ form an exciplex, with the negative charge localized primarily on Chl_2_ (Cherepanov et al., 2020, 2021). While there are differences that remain in the field, and the mechanism may vary based on experimental conditions and across organisms, it should be noted that similar models have been proposed in other Type I RCs (Kojima et al., 2020; Song et al., 2021).

While the overall mechanism remains under study, the lifetime of the various electron-transfer steps of PS I are reasonably well understood. Exciton migration from the antenna system to the trap appears to be the limiting step in charge separation, occurring with a lifetime of ∼ 5 ps (Wasielewski et al., 1987; Shelaev et al., 2010). However, charge separation is known to occur within 100 fs of photoexcitation of the trap (Shelaev et al., 2010). It is upon charge separation that the electron-transfer pathway bifurcates between the A- and B-branches, forming the P_700_^⋅+^A_0A_^⋅–^ or P_700_^⋅+^A_0B_^⋅–^ state. Unlike Type II RCs, each branch is utilized, albeit unequally, with the A-branch being favored in cyanobacteria by a factor of two in comparison with the B-branch (Agalarov and Brettel, 2003; Poluektov et al., 2005). Electron transfer then proceeds to the A_1A_ and A_1B_ cofactors within ∼ 30–50 ps (Brettel, 1997; Itoh et al., 2001), and then within 20 or 200 ns, respectively (Agalarov and Brettel, 2003; Kurashov et al., 2018) to the inter-polypeptide [4Fe–4S] cluster, F_X_, where the A- and B-branches are known to converge (Figure 4B). Subsequently, electron transfer occurs linearly though the F_A_ and then F_B_ clusters (Díaz-Quintana et al., 1998) with a lifetime of ∼ 200 ns, after which the electron is transferred to a soluble electron acceptor, ferredoxin (Mondal and Bruce, 2018) or flavodoxin (Pierella Karlusich and Carrillo, 2017), for downstream processes. For a detailed analysis of the electron-transfer and charge-recombination lifetimes in PS I, please see (Kurashov et al., 2018).

It is important to understand the structural and electronic factors that influence the redox and kinetic properties of the primary donor, P_700_, as it is critical for light-driven electron transfer in PS I. P_700_ is a dimer of a Chl *a*’ and Chl *a* molecules, Chl_1A_ and Chl_1B_, where Chl *a*’ is the 13^2^ epimer of Chl *a* (Kobayashi, 1996; Webber and Lubitz, 2001; Figure 4C). Of all of the primary donors that are discussed in this review, P_700_ appears to have the most extensive and asymmetric protein-cofactor interactions based on the X-ray crystal structure (Jordan et al., 2001). Perhaps an obvious indication is the striking asymmetry of the hydrogen (H)-bonding environment of the two Chl molecules, Chl_1A_ and Chl_1B_, that comprise the primary donor. As can be seen in Figure 4C, Chl_1A_ has three hydrogen bonds, provided by the Tyr735_PsaA_, Tyr603_PsaA_, and Thr743_PsaA_ residues, with a hydrogen H-bonding distance of 2.8, 3.3, and 3.1 Å, respectively. Additionally, there is a water molecule within 3.3 Å of Chl_1A_, which is not present near Chl_1B_. While it is possible that the hydrogen bonds may facilitate selective binding of the Chl *a*’ epimer, these are also likely to serve as a means to alter the redox potential of Chl_1A_ relative to Chl_1B_, providing strict control over the distribution of electron density. The hydrogen bonds do not appear to be the only significant deviation in the protein matrix that could influence asymmetry between Chl_1A_ and Chl_1B_, as three Leu residues (Leu630_PsaB_, Leu650_PsaA_, and Leu654_PsaA_) preferentially interact with Chl_1B_, although Leu654_Psa_ and Leu630_PsaB_ are found in the non-overlapping regions of the rings. Beyond this, both contain axially ligated His residues, which should not contribute to any asymmetrical effects.

Alterations to the primary donor, P_700_, of PS I from C*hlamydomonas reinhardtii* with the axial ligands, His656_PsaB_ and His676_PsaA_, have demonstrated profound effects on its properties. Initial studies, where the His656_PsaB_ residue was replaced by Asn or Ser, displayed a shift in the mid-point potential of P_700_ by +40 mV, with a corresponding alteration in its spectral features. While such changes are to be expected, electron-nuclear double resonance (ENDOR) spectroscopy of P_700_^⋅+^ in the genetic variants revealed an increase in electron spin density at the methyl group in position 12 by ∼ 20%, suggesting a significant effect on the electron delocalization over the two halves of the dimer (Webber et al., 1996). Subsequently, additional variants were studied that included changes to the axial ligand of Chl_1A_ on the A-side, His676_PsaA_, and non-polar, acidic, basic, and uncharged residues in the vicinity. Changes to the redox properties of P_700_ displayed an expected pattern, where soft base ligands, such as, the S atom of a Cys residue, had the most profound effect on the redox potential, shifting the potential by up to ∼ + 140 mV. The effects on the electronic properties of P_700_^⋅+^ were subtle and highly asymmetric.

**FIGURE 5:**
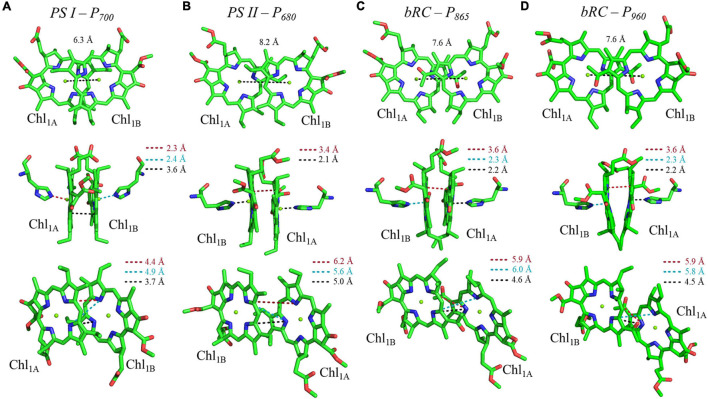
Analysis of inter-cofactor distances and relative orientation of primary donors from the heterodimeric RCs: **(A)** Photosystem I, **(B)** Photosystem II, and the bacterial RC from **(C)** *Rba. sphaeroides* and **(D)** *Rps. viridis*.

**FIGURE 6:**
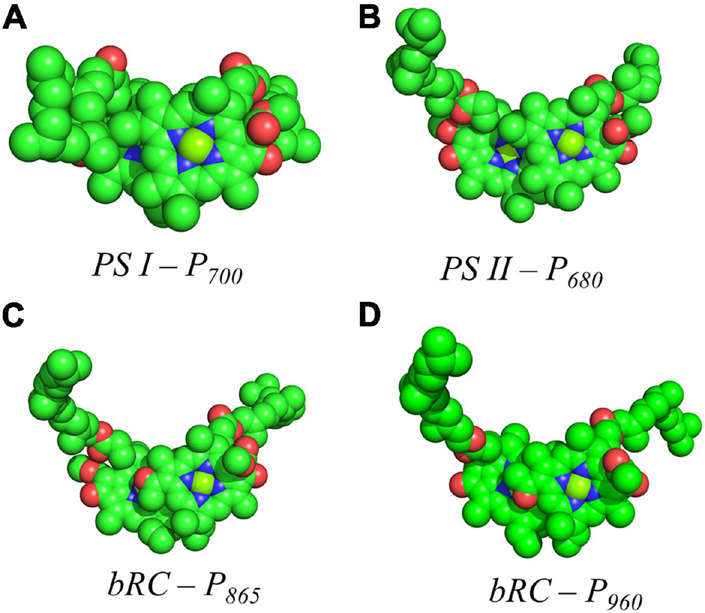
Space filling models of **(A)** P_700_ from PS I, **(B)** P_680_ from PS II, **(C)** P_865_ from the bRC of *Rba. Sphaeroides* and **(D)** P_960_ from the bRC of *Rps. Viridis*.

Shown in Figure 5A are the inter-cofactor distances and orientation of the Chl_1A/1B_ molecules of P_700_. P_700_ has perhaps the most archetypal parallel orientation of the two Chl macrocycles of Chl_1A_ and Chl_1B_ in the dimer, with the distance between the macrocycles varying from 3.5–3.6 Å. There is significant overlap of the ring planes of the Chl_1A_ and Chl_1B_ macrocycles, as the center-to-center distance between the two Mg^2+^ ions is 6.3 Å. Much like every other primary donor, the most prominent overlap occurs over the pyrrole group of the N^3^ atom, with a small overlap of the pyrrole of N^2^. The space-filling model in Figure 6A shows the extent of the overlap between the two macrocycles. Because of the significant overlap, the nearest ring nitrogen atoms are amongst the closest amongst all of the RCs discussed here. The most relevant are the N^3A^–N^3B^, N^2A^–N^2B^, and N^3A^–N^2B^ distances of 3.7, 4.4, and 4.9 Å, respectively. These values are shown in Table 1 for comparison with analogous distances in other RCs. Figure 6A also highlights the typical orientation of the phytol tail of P_700_ in comparison with that of other Type I RCs, i.e., a configuration that is tight to the central rings of the macrocycles with a small bend influenced by the presence of a water molecule.

**TABLE 1:** Distance parameters of the primary donors of heterodimeric and homodimeric RCs.

Heterodimeric RCs						
Primary Donor	Mg^2+^–Mg^2+^	Ring–Ring	Nearest Nitrogens*			
			N^3A^–N^3B^	N^2A^–N^2B^	N^3A^–N^2B^	N^2A^–N^3B^
P_700_	6.3 Å	3.4–3.6 Å	3.7 Å	4.4 Å	4.9 Å	4.9 Å
P_680_	8.2 Å	3.4–3.6 Å	5.0 Å	6.2 Å	5.6 Å	6.8 Å
P_865_	7.6 Å	3.6–3.8 Å	4.6 Å	6.3 Å	6.0 Å	5.9 Å
P_960_	7.6 Å	3.6–3.8 Å	4.5 Å	6.2 Å	5.9 Å	5.8 Å
**Homodimeric RCs**						
**Primary Donor**	**Mg^2+^–Mg^2+^**	**Ring–Ring**	**Nearest Nitrogens**			
			**N^3^–N^3^**	**N^2^–N^2^**	**N^3^–N^2^**	**N^2^–N^3^**

P_800_	5.7 Å	3.1–3.5 Å	3.1 Å	4.7 Å	4.7 Å	4.7 Å
P_840_	6.4 Å	3.3–3.6 Å	3.9 Å	5.7 Å	5.5 Å	5.4 Å

*The labels A and B refer to BChl_1*A*_ and BChl_1B_ for P_680_, P_960_, and P_865_.

#### The Primary Donor, P_680_, of Photosystem II

This section is focused on the Type II heterodimeric RC, photosystem II (PS II) [reviewed in Vinyard et al. (2013) and Lakshmi et al. (2014)]. The primary electron-transfer cofactors of PS II are present in the core polypeptide subunits, D1 and D2, and encompassed by ∼ 22–23 smaller polypeptides, whose identity varies among differing organisms. The primary electron-transfer pathway of PS II is composed of the following cofactors that are common to heterodimeric Type II RCs: four pseudo-symmetric Chl *a* molecules, two pheophytins (Pheo_A_ and Pheo_B_) and two quinones (Q_A_ and Q_B_) (Figures 4D,E). In contrast to other Type II RCs, the electron-transfer pathways also contain two redox-active tyrosine residues, Y_D_ and Y_Z_, and a unique tetranuclear manganese calcium-oxo (Mn_4_Ca-oxo) cluster, which is the well-known water-splitting catalytic moiety of PS II. The redox properties of the primary donor, P_680_, further differentiate PS II from other Type I and Type II RCs. The primary donor, P_680_, of PS II is by far the most oxidizing of any RC discovered thus far, with an *E*_*m*_ of ∼ 1,200 mV (Rappaport et al., 2002; Ishikita et al., 2005, 2006; Figure 3). In fact, it is one of the most oxidizing species that is found in nature. The evolutionary reason for this is clear, as P_680_ needs to be a strong oxidant in order to allow for successive oxidations of the Mn_4_Ca-oxo cluster that leads to the catalytic conversion of two substrate water molecules into dioxygen. The extremely high oxidizing potential of P_680_ showcases the amazing ability of nature in modulating the redox potential of identical or highly similar cofactors for different purposes (Ishikita et al., 2006).

Once again, there are various proposals in literature on the location of the primary charge separation of PS II, but recent findings suggest that charge separation originates at the ‘accessory’ Chl, termed Chl_2A_ (Groot et al., 2005; Holzwarth et al., 2006b; Romero et al., 2010; Duan et al., 2017; Sirohiwal et al., 2020; Tamura et al., 2020). Theoretical modeling of site energies has indicated that contrary to previous assumptions, Chl_2A_ has the lowest energy of all of the initial Chls. The reason for this deviation from other Type II heterodimeric RCs (see below) is attributed to the need to incorporate the Mn_4_Ca-oxo cluster used in water splitting (Tamura et al., 2020). In other studies, Stark spectroscopy was used to observe multiple mixed exciton charge-transfer states: (Chl_1B_^δ+^Chl_1A_^δ–^Chl_2A_)^∗^, (Chl_2A_^δ+^Pheo_D_^δ–^)^∗^, and (Chl_1B_^+^Chl_1A_^–^)^δ*^, suggesting a highly electronically coupled model. Interestingly, multiple empirical methods have found two events associated with charge separation, at 100–400 fs and ∼1.8 ps (Romero et al., 2010; Duan et al., 2017). Regardless of the mechanism, within ∼ 1.5 ps of photoexcitation of the trap, an electron is transferred to Pheo_A_, forming the charge-separated [P_680_^⋅+^Pheo_A_^⋅–^] state. Unlike PS I, electron transfer within PS II is branch specific, only occurring through the A-side involving the cofactors associated with the D1 polypeptide subunit (with the exception of the primary quinone, Q_A_, that is bound in the D2 polypeptide). The stabilization of the relatively short-lived P_680_^⋅+^Pheo_A_^⋅–^ state is achieved through rapid forward electron transfer to the primary quinone acceptor, Q_A_, within 200 ps and then to the secondary quinone, Q_B_, within ∼ 200–400 μs. Much like the bRC, Q_B_ is doubly reduced and doubly protonated after two turnovers of the RC, forming the labile quinol, Q_B_H_2_, with the second electron transfer step occurring notably slower than the first one (∼500–800 μs) (Bowes et al., 1980; Robinson and Crofts, 1983; de Wijn and van Gorkom, 2001; Sauer and Yachandra, 2004). As previously mentioned, the terminal quinone acceptors are a hallmark of Type II RCs as are the [4Fe–4S] clusters of the Type I RC, PS I. Meanwhile, on the acceptor side, the hole on P_680_^⋅+^ is reduced by the redox-active tyrosine, Y_Z_, which, in turn, is re-reduced by the Mn_4_Ca-oxo cluster. Two turnovers of PS II are required for the complete reduction of Q_B_ and a total of four turnovers results in the oxidation of two molecules of substrate water to dioxygen at the catalytic Mn_4_Ca-oxo cluster (Vinyard et al., 2013).

The primary donor of PS II, P_680_, is a dimer of Chl *a* molecules, Chl_1A_ and Chl_1B_. In contrast with P_700_, which displays an asymmetry of interactions with the protein matrix, P_680_ of shows less asymmetry in the protein matrix for each Chl *a* molecule. P_680_ contains one hydrogen bond at each Chl *a* provided by the Ser282_D2_ residue and a water molecule at Chl_1A_ and Chl_1B_, respectively (Figure 4F). Moreover, the axial ligand to each Chl *a* is a His residue, His197_D1_ and His198_D2_, where the His197_D1_ residue also serves to bind a water molecule in proximity of the Chl_1A_ cofactor. It should be noted that the importance of the His198_D2_ residue is in question and may well be species-dependent. While the alteration of the His198_D2_ residue in PS II from *Synechocystis* sp. PCC 6803 displayed changes in the spectral profile of P_680_ with a corresponding drop in the redox potential of ∼ 80 mV (Diner et al., 2001), analogous genetic variants of His198_D2_ in PS II from *Thermosynechococcus elongatus* displayed no discernable effects on the spectral, redox, or kinetic properties of P_680_ (Sugiura et al., 2008). Aside from the H-bonding interactions, the only other deviation between the environment of Chl_1A_ and Chl_1B_ of P_680_ are the residues Leu182_D2_ (near Chl_1B_) and Met183_D1_ (near Chl_1B_), in the non-overlapping region of the Chl *a* macrocycles.

The primary donor, P_680_, further differentiates itself from P_700_ in terms of the inter-cofactor distances and relative orientation of the Chl *a* molecules. While the macrocycles adopt a parallel orientation with a similar distance between the ring planes of Chl_1A_ and Chl_1B_ of 3.4–3.6 Å, the distance between the Mg^2+^ ions increases to 8.2 Å in P_680_. This results in minimal overlap of Chl_1A_ and Chl_1B_, which is evident only in part of the N^3^ pyrrole group (Figure 5B, Top Panel). The limited overlap of the ring planes is further manifest as an increased distance between the nearest nitrogen atoms of Chl_1A_ and Chl_1B_. The distance for the three nearest nitrogen atoms are: N^3D1^–N^3D2^ (5.0 Å), N^3D1^–N^2D2^ (5.6 Å), and N^2D1^–N^2D2^ (6.2 Å) (Figure 5B, bottom panel). This reveals that not only is there an increase in the distance between proximal nitrogen atoms of Chl_1A_ and Chl_1B_, but the longest distance of the three nitrogen atoms is observed in N^2D1^–N^2D2^ (as opposed to N^3A^–N^2B^ for P_700_). This suggests that the macrocycles exhibit a small degree of ‘outward’ rotation relative to each other. However, the orientation of the tail of P_680_ is typical of other Type II RCs, where it extends outward toward the stromal side of the protein. The space-filling model shown in Figure 6B provides a visual representation of the change in ring overlap, slight outward rotation, and the change of the tail orientation of P_680_.

#### The Primary Donor, P_865_ and P_960_, of the Bacterial Reaction Centers

**FIGURE 7:**
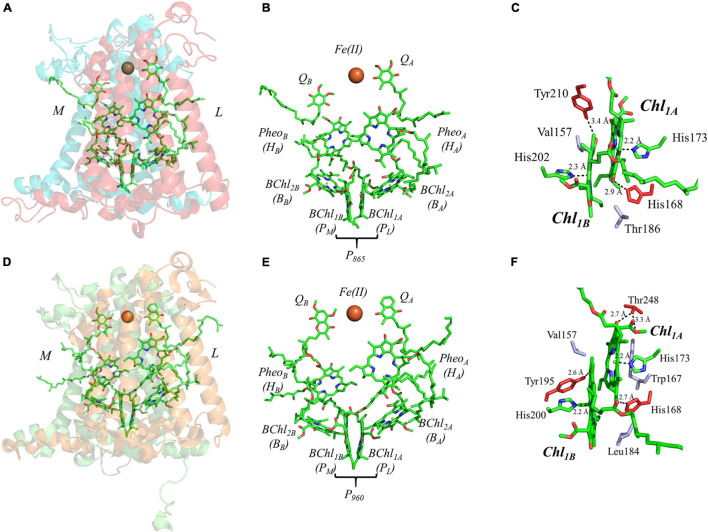
**(A)** The core subunits (M, blue; and L, red) and cofactors of the bRC from *Rba. sphaeroides* as observed by X-ray crystallography (PDB ID: 1aij), **(B)** the cofactors that participate in the primary electron transfer pathway of the bRC from *Rba. sphaeroides* and **(C)** the binding pocket of the primary donor, P_865_. **(D)** The core subunits (M, blue; and L, red) and cofactors of the bRC from *Rps. viridis* as observed by X-ray crystallography (PDB ID: 2jbl), **(E)** the cofactors that participate in the primary electron transfer pathway of the bRC from *Rps. viridis* and **(F)** the binding pocket of the primary donor, P_960_. Commonly used labels are in parentheses. Part **(C,F)** emphasize the prominent protein-matrix effects of the primary donor of the bRC from *Rba. sphaeroides* and *Rps. viridis*, respectively, where the residues that are hydrogen bonded to the primary donors are shown in red, and nearby non-polar or π-stacked residues are colored in gray.

The final set of heterodimeric primary donors is that of the bRCs from *Rba. sphaeroides* and *Rps. viridis* (more recently known as *Bl. viridis*) [reviewed in Leonova et al. (2011) and Savikhin and Jankowiak (2014)]. The determination of the X-ray crystal structure of these RCs dates back to several decades. Indeed, the first X-ray crystal structure of a membrane protein ever obtained was from *Rps. viridis*, for which Hartmut Michel, Robert Huber, and Johann Deisenhofer were awarded the Nobel Prize in chemistry in 1988 (Deisenhofer et al., 1985). In contrast with the other heterodimeric RCs, the bRC is relatively simple, being composed of only three polypeptide subunits, L, M, and H. An additional subunit containing four cytochrome *c* polypeptides is present in *Rps. viridis*, but the electron-transfer cofactors are entirely contained within the L and M subunits of the bRC. Similar to PS II, the electron transfer chain of the bRC is comprised of four bacteriochlorophylls (BChl), two bacteriopheophytins (BPheo_A_ and BPheo_B_) and two quinones (Q_A_ and Q_B_) (Figures 7A,B,D,E).

The predominant model of charge separation in the bRC includes the initiation of electron transfer at the primary donor (Niedringhaus et al., 2018), P_865_ and P_960_, in *Rba. sphaeroides* and *Rps. viridis*, respectively. Theoretical studies have even suggested that charge separation can occur in the primary donor between BChl_1A_ and BChl_1B_ itself (Xu et al., 2002). However, Huang and coworkers used polarization selective spectroscopic methods to demonstrate that the excitation of BChl_2_ can lead to two sub-populations, wherein there is rapid formation of BChl_2A_^⋅+^Pheo_A_^⋅–^ in approximately half of the RCs, and the excitation migrates to P_865_ in the remaining RCs (Huang et al., 2012). The photoexcitation of the primary donor results in charge separation within 3–5 ps in *Rba. sphaeroides* (Martin et al., 1986; Holzapfel et al., 1989; Lauterwasser et al., 1991; Peloquin et al., 1994; Holzwarth and Müller, 1996) and a slightly faster rate of ∼ 2 ps in *Rps. viridis* (Wasielewski and Tiede, 1986; Huppman et al., 2002). The rapid formation of the Pheo_A_^⋅–^ state is unexpected given its long distance from the primary donor. Hence, it was proposed that the accessory BChl, BChl_2_, may play a role in charge separation. Through a combination of transient absorption spectroscopy (Arlt et al., 1993; van Stokkum et al., 1997), ultrafast mid-IR spectroscopy (Pawlowicz et al., 2008), mutagenesis (Shkuropatov and Shuvalov, 1993; Heller et al., 1996; Kennis et al., 1997; Roberts et al., 2001), and cofactor replacement experiments (Kirmaier et al., 1995a,b), the presence of BChl_2_^⋅–^ was indeed detected and it was found to play an integral role in electron transfer. However, the first stable electron transfer intermediate is largely considered to be Pheo_A_^⋅–^, after which the electron is transferred to the primary quinone, Q_A_, in ∼ 200 ps. The reduction of Q_A_, and the subsequent change in the presence of an electric field causes a structural perturbation to the solvation (and ultimately the protein) structure (Arata and Parson, 1981; Kleinfeld et al., 1984b; McMahon et al., 1998), which allows for the final step of electron transfer and the reduction of the secondary quinone, Q_B_. Similar to PS II, two turnovers of the bRC are required for the complete reduction of Q_B_, which functions as a two-electron/two-proton acceptor, forming the quinol, Q_B_H_2_ (Wraight, 2004).

The primary donor of the bRC is a dimer of two BChl molecules that are bound by the L and M polypeptide subunits. It is interesting that the identity of the BChls, and thus the spectral features of the primary donors, P_865_ and P_960_, of the bRC from *Rba. sphaeroides* and *Rps. viridis* are different. In *Rba. sphaeroides*, the primary donor, P_865_, is a dimer of BChl *a* molecules, while P_960_ of *Rps. viridis* is a dimer of BChl *b* molecules. In this section, we will focus on both bRCs as the differences between the two are minor. The binding site of P_865_ and P_960_ suggests a moderately asymmetric H-bonding environment that could influence the relative redox potential of each primary donor. As shown in Figures 7C,F, there is a hydrogen bond to the BChl molecules of P_865_ and P_960_ from the Tyr210_L_ and Tyr195_L_ residue that are within 3.4 and 2.6 Å, of BChl_1B_, respectively, and the His168_L_ that is within 2.9 and 2.7 Å of BChl_1A_ in P_865_ and P_960_, respectively. There is an additional residue that provides a hydrogen bond to BChl_1A_ in P_960_, Thr248_L_, with the hydroxyl and carboxyl group at a distance of 2.7 and 3.3 Å, respectively. Additional differences between the two bRCs include the residues that are located in the non-overlapping region of the BChl rings. In P_960_, there are two non-polar residues, Val157_L_ and Leu184_M_, whereas, in P_865_ there is a non-polar, Val157_L_, and an unusual polar residue, Thr186_M_. The axial ligands of all of the BChl molecules, however, remain similar to all of the other primary donors, with His202_M_ (P_865_)/His200_M_ (P_960_) and His173_L_ ligating BChl_1B_ and BChl_1A_, respectively.

Further analysis of the inter-cofactor distances and relative orientation of the BChl macrocycles of P_865_ of the bRC from *Rba. sphaeroides* reveals a structure that is similar to P_680_ of PS II, albeit with unusual features. A view of the macrocycle plane (Figure 5C, Top Panel) indicates an overlapping structure that is similar to PS II, whereby the overlap of the two BChl rings is nearly exclusively on the pyrrole associated with N^3^. The Mg–Mg distance of P_865_ is 7.6 Å, which is in between PS II (8.2 Å) and PS I (6.3 Å); however, there is significantly more overlap than exists in P_680_. But perhaps the most defining feature of the BChl dimer of P_865_ is in how it deviates from a parallel orientation of the ring planes. The macrocycles form a ‘V’ shape when viewed parallel to the ring plane, pointing toward the luminal side of the protein. While most prominently seen in *Rps. viridis*, it is also observed in *Rba. sphaeroides* (Figures 5C,D). As expected, this results in a change of the distance between the nearest nitrogen atoms of the macrocycles. While the N^3^ nitrogen atoms remain the closest at 4.6 Å and the N^3^_M_–N^2^_L_ distance is next at 6.0 and 5.9 Å in P_865_ and P_960_, respectively, N^2^_M_ is located at a distance of 5.9 and 5.8 Å from N^3^_L_ in P_865_ and P_960_, respectively, which is different from the pattern that is observed in other RCs. The space-filling model P_865_ and P_960_ shown in Figures 6C,D displays the macrocycle overlap and tail orientation that is common to other Type II RCs.

### Geometric Structure of the Primary Donor of Homodimeric Reaction Centers

#### The Primary Donor, P_800_, of *Heliobacterium modesticaldum*

**FIGURE 8:**
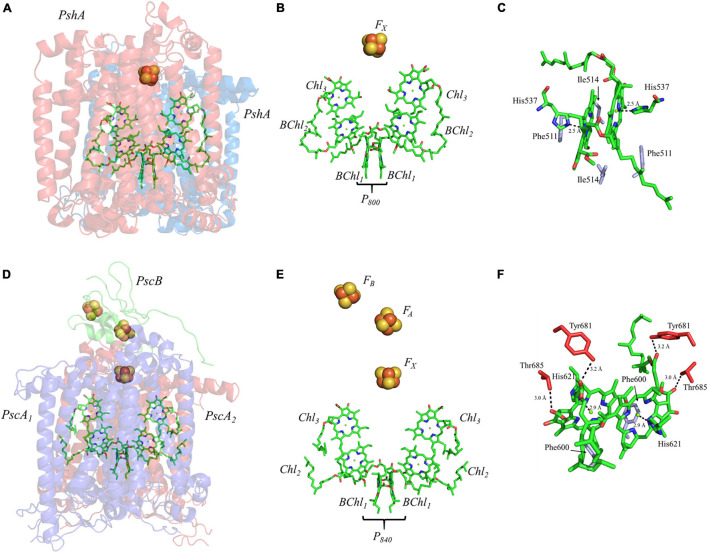
**(A)** The core subunits (PshA, Blue and Red) and cofactors of the HbRC from *H. modesticaldum* as observed by X-ray crystallography (PDB ID: 5v8k), **(B)** the cofactors that participate in the primary electron transfer pathway of the HbRC and **(C)** the binding pocket of the primary donor, P_800_. **(D)** The core subunits (PscA_1_, blue; PscA_2_, red; and PscB, green) and cofactors GsbRC from *C. tepidum* as observed by cryo-electron microscopy (PDB ID: 6m32), **(E)** the cofactors that participate in the primary electron transfer pathway of the GsbRC and **(F)** the binding pocket of the primary donor, P_840_. The residues in part **(C,F)** that are providing hydrogen bonds are shown in red, and nearby non-polar or π-stacked residues are colored in gray.

The homodimeric RC from *H. modesticaldum*, HbRC, is a simplified complex which is often considered an exemplar of ancestral Type I RCs (Ferlez et al., 2016; Orf et al., 2018). It has long been known that the HbRC is composed of a dimer of PshA polypeptide subunits, but it was not until the availability of the X-ray crystal structure that another identical pair of polypeptide subunits, PshX, were found on the periphery of each PshA polypeptide subunit (Gisriel et al., 2017; Figure 8A). The electron-transfer cofactors are completely housed in the PshA polypeptide core, and are comprised of four symmetric BChl *g* molecules, two 8^1^-OH Chl *a* molecules, and a single inter-polypeptide [4Fe–4S] cluster, F_X_ (Figure 8B). Note the lack of the two quinones and two additional [4Fe–4S] clusters bound by a small soluble ferredoxin-like protein present in the other Type I RC, PS I, discussed above (Romberger and Golbeck, 2012).

The mechanism(s) of primary charge separation in the HbRC has been the focus of several recent studies. The unusual pigment composition of the HbRC, where multiple types of (B)Chl molecules are involved in electron transfer, lends itself particularly well to the direct observation of the transient excited states using optical spectroscopic methods. Ultrafast pump-probe spectroscopy was initially employed to study energy transfer amongst the various pools of BChl molecules as well as the initial states of charge separation, suggesting a highly coupled system of early BChl molecules (Kojima et al., 2020). Subsequently, global analysis of two-dimensional electronic spectra suggested multiple pathways of charge separation. The direct photoexcitation of the trap was suggested to generate the excited state [BChl_2_/Chl_3_]^∗^, which then extended to [P_800_/BChl_2_/Chl_3_]^∗^ in ∼ 90 fs and led to the formation of the first charge-separated state, [P_800_/BChl_2_]^⋅–^Chl_3_^⋅+^, within 900 fs (Song et al., 2021). Alternately, the excitation of the antenna system led to the formation of the initial charge-separated state, [P_800_/BChl_2_]^⋅–^Chl_3_^⋅+^, within 2.2 ps. Regardless of which mechanism is involved, the final charge separated state, P_800_^⋅+^A_0_^⋅–^, was formed within 20–25 ps, which is in agreement with previous observations (Chauvet et al., 2013; Kojima et al., 2020). Once the stable P_800_^⋅+^A_0_^⋅–^ state is formed in the HbRC, the electron is transferred to the F_X_ cluster in 600–800 ps (Nuijs et al., 1985a; Chauvet et al., 2013).

Much like PS I, the primary donor of the HbRC, P_800_, is composed of an epimer of the primary pigment of the RC, which is BChl *g*’ (Kobayashi et al., 1991). The P_800_ dimer may be the most interesting of all the cases discussed here, not because it contains extensive protein-matrix effects like those observed for P_700_, but rather due to the lack of apparent protein matrix effects from the surrounding environment. While FTIR spectroscopy had previously suggested that a cysteine residue, either Cys469 or Cys601, may be hydrogen bonded to the primary donor itself (Noguchi et al., 1997), the high-resolution X-ray crystal structure of the HbRC has revealed that this is not the case (Gisriel et al., 2017). Instead, the differential signal at 2550/2560 cm^–1^ that was observed in the FTIR spectra was likely due to Cys601, a residue that is in close proximity to the axial His ligands, His537, of P_800_. Indeed, there are no apparent hydrogen bonds that have been observed in either of the BChl *g*’ molecules of the HbRC (Figure 8C). Also lacking are any effects from neighboring water molecules. The only observable residues are Ile514 that resides in the non-overlapping region of the BChl *g*’ macrocycles and a nearby Phe511 residue within 4.4 Å that is oriented in a pseudo π-stacked fashion.

**FIGURE 9:**
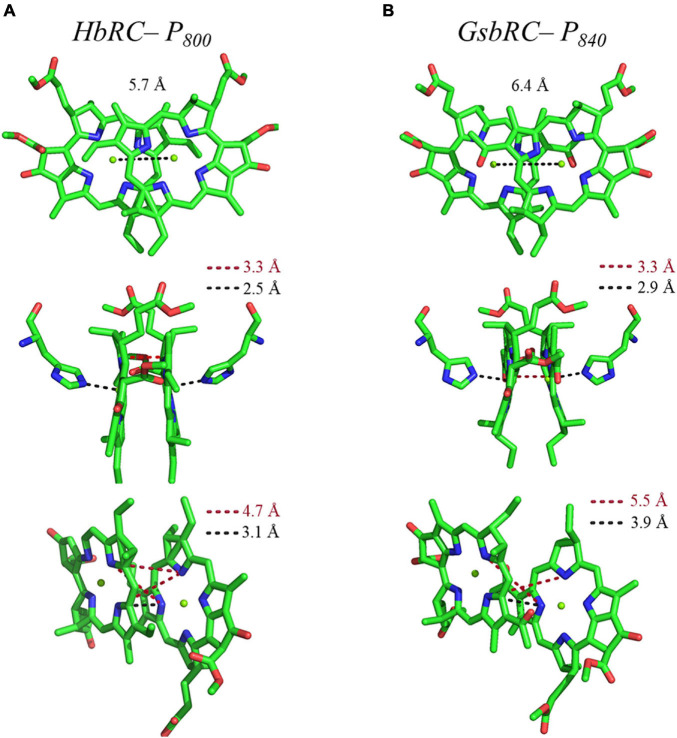
Analysis of inter-cofactor distances and relative orientation of primary donors from the homodimeric RCs: **(A)** P_800_ from the HbRC, and **(B)** P_840_ from the GsbRC.

**FIGURE 10:**
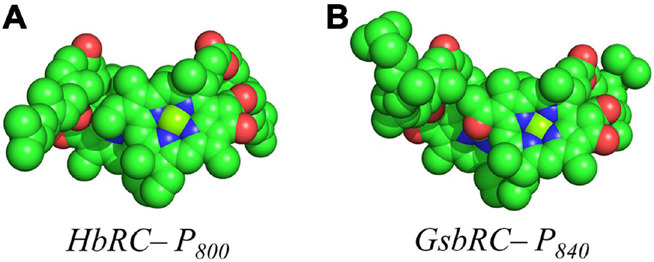
Space filling models of **(A)** P_800_ from the HbRC and **(B)** P_840_ from the GsbRC.

Several facets of the inter-cofactor distances and relative macrocycle orientation further showcase the unique nature of the BChl *g*’ dimer of P_800_. The Mg–Mg distance is the shortest of all known RCs, at 5.7 Å, which results in a significant amount overlap of the BChl *g*’ macrocycles (Figure 9A). Moreover, while the macrocycles maintain a roughly parallel orientation, there is a minor bend in each BChl, which causes the distance between the rings to vary from 3.1 to ∼ 3.5 Å, the former being the shortest distance between ring planes seen thus far. An interesting artifact of the macrocycle structure is reflected in the distance between the nearest nitrogen atoms of the rings. Not only is N^3^–N^3^ distance of 3.1 Å representative of the closest point between the two macrocycles, the distance between N^2^–N^2^ and N^3^–N^2^ for each ring is identical at 4.7 Å (the distance parameters for homodimeric RCs are summarized in Table 1). This suggests a highly coupled dimer resulting in nearly symmetric electron density on each macrocycle. The extensive overlap of the two macrocycles of P_800_ is evident in the space-filling model shown in Figure 10A, with a tail orientation typical of Type I RCs.

#### The Primary Donor, P_840_, of *Chlorobaculum tepidum*

The RC from the green sulfur bacterium *C. tepidum*, GsbRC, is a homodimer of the membrane-bound PscA polypeptide subunit (Figures 8D,E), which is encoded by a single gene, *pscA* (Hager-Braun et al., 1999; Hauska et al., 2001). The GsbRC core also includes PscB, a polypeptide analogous to PsaC in PS I, however, this polypeptide subunit is less tightly bound than PsaC and can be removed with detergent treatment (Miller et al., 1992; Schmidt et al., 2000) or at relatively low salt concentration (Miller et al., 1992; Jagannathan and Golbeck, 2008). While the polypeptide core and accompanying cofactors of the GsbRC should be considered a homodimer, the symmetry is broken by the PscB polypeptide, whose arbitrary orientation (in conjunction with PscD) directs the binding of the FMO-1, -2, and -3 proteins on the cytoplasmic side of the membrane (Chen et al., 2020). The electron-transfer cofactors reside within the homodimeric core and are comprised of two BChl molecules that constitute the primary donor, P_840_, four Chl *a* molecules, and three [4Fe–4S] clusters, F_X_, F_A_, and F_B_. Similar to the HbRC, note the absence of any quinones between Chl_3_ and the [4Fe–4S] clusters. For a detailed review of the GsbRC, please see (Hauska et al., 2001).

Charge separation in the GsbRC appears to follow a pattern similar to that of other Type I RCs. Time-resolved transient absorption spectroscopy at various temperatures has suggested that a highly electronically coupled system of (B)Chls are responsible for energy trapping and primary charge separation (Neerken et al., 1998, 1999). It should be noted that similar data is not available for the homodimeric HbRC, hence, a detailed comparison of the mechanism is not possible here. Regardless, photoexcitation of the trap appears to be complete within 2 ps, allowing charge separation to occur within 25 ps (Francke et al., 1996; Neerken et al., 1998, 1999), a lifetime that is nearly identical to the HbRC. Optical spectroscopy on the picosecond time scale showed that subsequent electron transfer from A_0_^⋅–^ to F_X_ occurs within 600 ps (Nuijs et al., 1985b; Shuvalov et al., 1986), notably slower than A_0_^⋅–^ oxidation in PS I. Electron transfer then proceeds linearly through the two [4Fe–4S] clusters, F_A_ and F_B_, that are bound by the PscB subunit in <5 μs (Hauska et al., 2001).

Similar to PS I and HbRC, the primary donor of the GsbRC is comprised of a 13^2^-epimer, BChl *a*’ (Kobayashi et al., 2000). Examination of the primary donor of the GsbRC in Figure 8F reveals that the protein-matrix effects are much more pronounced than those of the analogous homodimeric RC, the HbRC, from *H. modesticaldum*. As discussed recently, the markedly lower resolution of the cryo-electron microscopy structure (as compared to other X-ray structures) suggests that assignments of protein matrix interactions should be considered tentative (Gisriel et al., 2021). There exists a total of four hydrogen bonds, two on each BChl macrocycle, provided by the Tyr681 and Thr685 residues (Figure 8F). However, as this is a homodimeric RC, it is expected that the effects of these interactions will impact the redox properties of each BChl *a* molecule equally and only serve to alter the redox potential of the primary donor, P_840_, as opposed to influencing the localization of the charge density. Another interesting feature of the protein matrix is the identity of the non-polar residues that fill the non-overlapping region of the macrocycles. While these tend to be amino acid residues with a short aliphatic side chain, such as Val and Ile in both heterodimeric and homodimeric RCs, there are aromatic residues in the GsbRC, *e.g.*, Phe600. The side chain of the Phe residues are orthogonal to the BChl *a* macrocycles, suggesting that there are no π-stacking interactions present. However, the aromatic residues are within a distance of 3.1 Å of the BChl *a* macrocycles and are likely to impact the redox properties of the P_840_ dimer.

The inter-cofactor distances and relative orientation of the BChl *a* macrocycles of the primary donor, P_840_, appear to be similar to P_700_ and P_800_ of PS I and the HbRC, respectively. The Mg–Mg distance of 6.4 Å for P_840_ (Figure 9B, Top Panel) is close to that of the P_700_ donor of PS I (6.3 Å), resulting in significant overlap of the two macrocycles, including both the pyrrole rings of N^3^ and to a lesser extent the pyrrole ring associated with N^2^. However, when viewed along the ring planes, it is clear that the macrocycles remain in a roughly parallel orientation, but adopt a non-planar or domed shape. While analogous to the HbRC, the deviation from planarity is much more extensive in P_840_. This bending results in a slight deviation in the identity of the nearest nitrogens of the macrocycles, where N^3^s remain proximal to each other at 3.9 Å, and both N^2^s are proximal to the opposing N^3^, at 5.4 and 5.5 Å. The space-filling model shows overlapping rings and a tail orientation that shares a high similarity to that of P_700_ (Figure 10B).

## Electronic Structure of the Primary Donor of Heterodimeric and Homodimeric Reaction Centers

### Continuous-Wave and Pulsed Electron Paramagnetic Resonance Spectroscopy Measurements

Continuous-wave (*CW*) and pulsed electron paramagnetic resonance (EPR) spectroscopy has been used for the study of the electronic structure and function of (B)Chl cofactors of RCs (Commoner et al., 1956; Käβ et al., 1995, 1996; Käβ and Lubitz, 1996). As described above, developments in protein crystallization and X-ray crystallography led to outstanding high-resolution structures of PS I, PS II, the bRC and HbRC from cyanobacteria, purple bacteria and heliobacteria, respectively (Stowell et al., 1997; Jordan et al., 2001; Umena et al., 2011; Gisriel et al., 2017). These advances have been complemented by other techniques, such as, cryo-electron microscopy, that provided the structure of the GsbRC from green sulfur bacteria (Chen et al., 2020). While these structures have delivered insight on the geometry and relative topology of the (B)Chl dimers in the neutral ground state, spectroscopic measurements have been instrumental in determining the electronic properties of the individual primary donors. *CW* and pulsed EPR methods have been especially well suited to probe the structure of the oxidized primary donors as the high sensitivity and specificity of the detection of unpaired electron spin(s) has helped overcome limitations of the large size of the RCs, and the use of powder samples has largely eliminated the need for soluble or crystalline material.

Photooxidation of the primary donor, P, generates a paramagnetic center with an unpaired electron spin, S, of 1/2 which makes it possible to probe the P^⋅+^ state by EPR spectroscopy. The EPR signal of P^⋅+^ is known to display a relatively small *g*-anisotropy, with a featureless resonance at X-band (9.6 GHz) frequency (Commoner et al., 1956; Norris et al., 1971). One of the earliest applications of *CW* EPR spectroscopy demonstrated that steady-state photoaccumulation of the primary donor cation, P_700_^⋅+^, of PS I (Commoner et al., 1956), resulted in a signal at a g value of 2.0025. Subsequently, Norris and coworkers showed that the EPR signal of P_700_^⋅+^ arises from a strongly excitonically coupled Chl *a* dimer (Norris et al., 1971; Davis et al., 1993; Mac et al., 1998; Käβ et al., 2001) with a line width that is narrower than that of monomeric Chl *a*^⋅+^ *in vitro* (Norris et al., 1971). This observation provided initial evidence for the delocalization of the unpaired electron spin across the dimeric Chl_1A/1B_ cofactors of P_700_^⋅+^ (Norris et al., 1971). The line width of the excitonically coupled dimeric Chl_1A/1B_ molecules of P_700_^⋅+^ is related to the width of a monomeric cation signal through the relationship, ΔH_N_ = 1/√N^⋅^ΔH_M_ (where ΔH_N_ and ΔH_M_ are the line width of the multimeric chlorophyll cation with spin delocalization, Chl_N_^⋅+^, and a monomeric Chl^⋅+^ cation, respectively). The presence of a narrower line width, and hence the possible delocalization of the unpaired electron spin, was also observed in the primary donor, P_680_^⋅+^ and P_865_^⋅+^, of PS II and the bRC from *Rba. sphaeroides*, respectively (van Gorkom et al., 1974; Davis et al., 1979).

Although the *g*-anisotropy of an oxidized primary donor, P^⋅+^, is not resolved at X-band (9.6 GHz) frequency, it is possible to observe the anisotropy at higher EPR frequencies (Webber and Lubitz, 2001). One of us has previously demonstrated that the *g*-anisotropy of P_700_^⋅+^ can be resolved at D-band (130 GHz) EPR frequency using perdeuterated PS I from *Synechococcus lividus* (Poluektov et al., 2002). These studies suggested that the *g*-anisotropy of P_700_^⋅+^ is smaller than that of monomeric Chl *a*^⋅+^ *in vitro*, which was explained by the delocalized electronic character of P_700_^⋅+^ or a heteromeric model of the primary donor. Using higher EPR frequencies, Angerhofer, Redding and coworkers determined the *g*-anisotropy of P_700_^⋅+^ of PS I from *Chlamydomonas reinhardtii* (Petrenko et al., 2004) and higher plants (Bratt et al., 1997, 2000), which suggested that the electron spin distribution of P_700_^⋅+^ may be more monomeric than dimeric. A similar comparison of the *g*-anisotropy of P_865_^⋅+^ of the bRC from *Rba. sphaeroides* with monomeric BChl *a*^⋅+^ *in vitro* also revealed little difference in the respective line widths (Burghaus et al., 1993). However, these results were in contrast with previous experimental and computational studies. Thus, it appeared that the magnitude of *g*-anisotropy of the primary donor cation, P^⋅+^, alone was not sufficient to provide definitive evidence on the extent of the delocalization of the electron spin over the (B)Chl molecules.

In principle, the direct measurement of magnetic hyperfine couplings between the unpaired electron spin and NMR-active nuclei could reveal the distribution of the electron spin density and hence, the electronic structure of the Chl_1A/1B_ dimer in the oxidized primary donor, P^⋅+^. However, it is not possible to measure the electron-nuclear hyperfine interactions using *CW* EPR spectroscopy due to the inhomogeneous broadening of the peaks in the spectrum. Therefore, hyperfine spectroscopy methods, such as ENDOR, electron-spin-echo envelope modulation (ESEEM) and two-dimensional (2D) hyperfine sub-level correlation (HYSCORE) spectroscopy, in conjunction with computational modeling, have been used to obtain information on the electronic structure of the oxidized primary donors (Britt, 1993; Deligiannakis et al., 2000; Lakshmi and Brudvig, 2000, 2001; Prisner et al., 2001; Harmer et al., 2009). The electron-nuclear hyperfine interactions determined by both experimental and computational methods have been used to identify the *CW* EPR signals, characterize the surrounding environment and coordination geometry, and quantitatively determine the hyperfine couplings and electron spin density distribution of the oxidized primary donors, P^⋅+^, in RCs. These measurements have included hyperfine interactions of the unpaired electron spin of the oxidized primary donor, P^⋅+^, with NMR-active nuclei, such as ^1^H, ^13^C, ^15^N and ^14^N of the (B)Chl molecules.

Electron-nuclear double resonance spectroscopy is a double resonance technique with reasonably high spectral resolution that has typically allowed for the measurement of small hyperfine couplings. Initial ENDOR spectroscopy studies of P^⋅+^ utilized *CW* radio-frequency irradiation, although this was replaced by pulsed methods, such as, Mims and Davies ENDOR, which have the advantage of more selective measurement of the nuclear hyperfine couplings (Hoffman, 2003; Kulik and Lubitz, 2009). More recently, ESEEM spectroscopy has been applied to the study of the oxidized primary donor, P^⋅+^, as it overcomes the inhomogeneous broadening of EPR resonances and provides access to unresolved electron-nuclear hyperfine couplings (Deligiannakis et al., 2000). The hyperfine couplings that are measured by ENDOR and ESEEM spectroscopy are orientation dependent, which means that information on the hyperfine anisotropy is lost in powder EPR samples with random orientations. Therefore, the hyperfine measurements of P^⋅+^ have also been performed on single-crystals of RCs or at high EPR frequencies, where the *g*-anisotropy of P^⋅+^ is better resolved making it possible to select for specific orientations of the molecules with respect to the applied magnetic field.

The nuclear transitions of multiple abundant spins, such as, ^1^H or ^14^N, in powder samples or frozen solutions have often been difficult to resolve by one-dimensional ENDOR and ESEEM spectroscopy due to spectral overcrowding of signals. Hence, we and others have been employing HYSCORE spectroscopy, which is a two-dimensional version of ESEEM, to obtain correlations between nuclear transitions to facilitate the detection and assignment of multiple hyperfine-coupled proton and nitrogen atoms of the oxidized primary donors, P^⋅+^, in two-dimensional frequency space (Höfer et al., 1986). All three of the hyperfine methods, ENDOR, ESEEM and HYSCORE, are highly sensitive as the electron spin-coupled nuclear transitions are monitored through the observation of a paramagnetic electron spin (Webber and Lubitz, 2001). Additionally, these methods are versatile as they can detect weak hyperfine couplings to less sensitive nuclei with smaller magnetic moments, such as, ^14^N and ^15^N atoms.

The hyperfine interactions of protons (^1^H), carbon (^13^C) and nitrogen (both ^14^N and isotope-labeled ^15^N) atoms of the oxidized primary donor, P_700_^⋅+^, P_865_^⋅+^, P_960_^⋅+^, P_680_^⋅+^, and P_840_^⋅+^ of the heterodimeric RCs, PS I, the bRC from *Rba. sphaeroides* and *Rps. viridis*, PS II and the homodimeric RCs, GsbRC from *Chlorobium limicola* and *Chloroacidobacterium thermophilum* (*Cab*. RC) (Charles et al., 2020), respectively, have been determined by pulsed EPR spectroscopy methods. As shown in Figures 4C,F, 7C,F, 8F, the primary donors of PS I, PS II, the bRCs and GbRC are comprised of a dimer of (B)Chl_1A_ and (B)Chl_1B_ molecules (Stowell et al., 1997; Jordan et al., 2001; Umena et al., 2011). Earliest estimates of the hyperfine coupling parameters of P_700_^⋅+^ of PS I were obtained by Dikanov, Astashkin and coworkers using ENDOR and ESEEM spectroscopy of the nitrogen atoms (both ^14^N and ^15^N) of the Chl *a* and Chl *a’* macrocycles interacting with the unpaired electron spin (Dikanov et al., 1983; Astashkin et al., 1987). It was observed that the unpaired electron spin of P_700_^⋅+^ displayed magnetic interactions with four nitrogen atoms, where two of the nitrogens were strongly hyperfine coupled with an isotropic hyperfine coupling, A_iso_, of ∼ 2 MHz, and two other nitrogens were weakly coupled with a much smaller A_iso_ of ∼ 0.2 MHz. While the early results were semi-qualitative, they established the feasibility of hyperfine measurements on P_700_^⋅+^ and highlighted the need for measurements that would yield higher spectral resolution.

Subsequently, the unpaired electron spin density distribution of the oxidized primary donors was determined by quantitative measurements of the hyperfine interactions of the protons and nitrogen atoms, which were compared with those of monomeric Chl *a*^⋅+^ and BChl *a*^⋅+^ *in vitro* (Astashkin et al., 1988). There is broad consensus in the field that the oxidized heterodimeric primary donors are comprised of a dimer of coupled (B)Chl molecules, albeit with small differences on the extent of asymmetry of the electron spin delocalization across the dimer. The asymmetric spin density distribution (ratio 3:1 to 5:1) of P_700_^⋅+^ has been observed by ^1^H, ^14^N and ^15^N ENDOR, HYSCORE and single crystal EPR and ENDOR measurements (Käβ et al., 1995; Käβ and Lubitz, 1996; Deligiannakis and Rutherford, 2001; Käβ et al., 2001; Webber and Lubitz, 2001; Chestnut et al., 2021). Recently, using ^14^N HYSCORE spectroscopy we conclusively established that there are at least four distinct ^14^N atoms (and likely more than four nitrogen atoms if we consider the possibility of overlapping cross-peaks in the spectrum) that are interacting with the unpaired electron spin of P_700_^⋅+^ (Gorka et al., 2021b). The isotropic hyperfine couplings, A_iso_, range from 1.4–2.8 MHz, indicating that the electron spin is distributed on at least four nitrogen atoms. In conjunction with previous and current findings, this indicates that P_700_^⋅+^ is comprised of a strongly electronically coupled Chl *a* dimer, where the unpaired electron spin density distribution is asymmetric over the two Chl a molecules (Käβ et al., 1995; Käβ and Lubitz, 1996; Deligiannakis and Rutherford, 2001; Käβ et al., 2001; Webber and Lubitz, 2001; Chestnut et al., 2021). A summary of the experimental hyperfine and quadrupolar couplings of the nitrogen atoms of P_700_^⋅+^ is presented in Table 2.

**TABLE 2:** Experimental ^14^N isotropic hyperfine couplings of the nitrogen atoms of P_700_^⋅+^, P_865_^⋅+^, and P_840_^⋅+^ of the GsbRC and *Cab*. RC.

Nitrogen	P_700_^⋅+^				P_865_^⋅+^	P_840_^⋅+^	P_840_^⋅+^
	A_iso_ [MHz]^a^	A_iso_ [MHz]^b^	A_iso_ [MHz]^c^	A_iso_ [MHz]^d^	A_iso_ [MHz]^e^	A_iso_ [MHz]^f^	A_iso_ [MHz]^g^
**N^I^**	2.8 ± 0.1	2.8 ± 0.4	2.13	2.36	2.65	1.14	0.90 ± 0.2
**N^II^**	2.4 ± 0.2	2.3 ± 0.3	2.06	1.95	2.48	1.35	1.10 ± 0.2
**N^III^**	1.77 ± 0.1	1.4 ± 0.2	1.95	1.13	2.11	1.35	0.90 ± 0.2
**N^IV^**	1.37 ± 0.2	1.2 ± 0.2	2.19	0.67	1.86	1.65	1.00 ± 0.2
**N^V^**		0.47 ± 0.07		0.25	0.52		
**N^VI^**		0.45 ± 0.07			0.48		
**N^VII^**					0.39		
**N^VIII^**					0.37		

*^a^Gorka et al. (2021b).*

*^b^Chestnut et al. (2021).*

*^c^Mac et al. (1998).*

*^d^Käβ et al. (1996).*

*^e^Käβ et al. (1995).*

*^f^Bratt et al. (1996).*

*^g^Charles et al. (2020).*

Early experimental measurements of P_865_^⋅+^ of the bRC from *Rba. sphaeroides* had determined two or four nitrogen hyperfine coupling tensors, which were on average smaller than those of the monomeric BChl *a*^⋅+^ *in vitro* by a factor of 2 (Lubitz et al., 1984). At the time, this seemed adequate as only four reduced hyperfine couplings were expected for the nitrogen atoms in a symmetric BChl dimer model of P_865_^⋅+^. Consequently, the first ENDOR and ESEEM spectroscopy studies were interpreted on the basis of an essentially symmetric spin density distribution across BChl_1A_ and BChl_1B_ of P_865_^⋅+^ (Lubitz et al., 1984; Lin et al., 1986; Astashkin et al., 1988). A detailed ^1^H ENDOR study performed on single crystals of the bRC from *Rba. sphaeroides* near room temperature showed that the 3:1 electron spin density distribution in favor of the Chl_1A_ (Lendzian et al., 1993), which was also observed for P_960_^⋅+^ of the bRC from *Rps. viridis* (Lendzian et al., 1988). This observation was further supported by the determination of ^15^N hyperfine couplings under similar conditions using ^15^N-labeled single crystals of the bRC (Lendzian et al., 1992). However, the complete hyperfine tensors were not determined in these experiments and hence, the assignment of the hyperfine couplings was only possible by comparison with molecular orbital calculations. These results were further confirmed by ESEEM measurements of P_865_^⋅+^ and P_960_^⋅+^ of the bRC from *Rba. sphaeroides* and *Rps. viridis* (Davis et al., 1993). Subsequently, a combined ESEEM and HYSCORE spectroscopy study of monomeric ^15^N-labeled BChl *a*^⋅+^ in solution and P_865_^⋅+^ in ^I5^N-labeled bRC from *Rba. sphaeroides* yielded a much higher asymmetry of the spin density ratio of approximately 5:1 (Käβ et al., 1995; Table 2).

Proton ENDOR and HYSCORE spectroscopy have also been employed for the study of the primary donor cation, P_680_^⋅+^, in core preparations of PS II containing the D1, D2 and cytochrome b_559_ polypeptides (Rigby et al., 1994a). The hyperfine parameters of the protons of methyl groups obtained from ^1^H ENDOR spectroscopy was used to calculate the electron spin density distribution of P_680_^⋅+^. Comparison of the hyperfine parameters and electron spin density distribution of P_680_^⋅+^ with the Chl *a*^⋅+^ monomer *in vitro* indicated an apparent reduction in the unpaired electron spin density for P_680_^⋅+^. Similar to P_700_^⋅+^ and P_865_^⋅+^, these studies suggested that P_680_^⋅+^ was a weakly coupled Chl_1A/1B_ dimer with 82% of the unpaired electron spin located on one of the chlorophyll molecules. Based on the experimental hyperfine parameters of P_700_^⋅+^, P_865_^⋅+^, P_960_^⋅+^, and P_680_^⋅+^, it is evident that the asymmetric spin density distribution that is observed does not appear to be caused by the structural difference of the (B)Chl molecules of P^⋅+^ in heterodimeric RCs, but by the interaction of the cofactors with the protein environment.

There have been far fewer reports on the primary donors of the homodimeric RC, HbRC and GsbRC. The oxidized primer donor, P_840_^⋅+^, of the GsbRC in *Chlorobium limicola* membranes was studied by ^1^H ENDOR and Triple electron-nuclear-nuclear spectroscopy (Rigby et al., 1994b). These studies showed that P_840_^⋅+^ is comprised of a BChl *a* dimer with a highly symmetrical distribution of electron spin density between BChl_1A_ and BChl_1B_. Moreover, Triple spectroscopy resolved the separate contributions of the two halves of the dimeric primary donor and revealed small deviations from a 1:1 electron spin density distribution. Subsequently, ^14^N ESEEM spectroscopy of the GsbRC in *Chlorobium limicola* membranes (Table 2) confirmed that the electron spin density distribution of P_840_^⋅+^ is shared equally between the two BChl *a* molecules (Bratt et al., 1996). In these studies, P_840_^⋅+^ was found to be closest yet to the symmetrical ‘dimer’ that was originally thought to exist in bRCs.

Most recently, we probed the oxidized primary donor, P_840_^⋅+^, of *Chloroacidobacterium* (*Cab*.) *thermophilum* with ^14^N and ^67^Zn HYSCORE spectroscopy (Charles et al., 2020). *Cab*. *thermophilum* is a microaerophilic, chlorophototrophic species in the phylum *Acidobacteria* that employs a homodimeric RC with BChl molecules. The *Cab*. RC is highly unusual, as pigment analyses have shown the presence of three (B)Chl molecules, BChl *a*_*P*_, Chl *a*_*PD*_, and Zn^2+^-BChl *a*_*P*_′, in the ratio 7.1:5.4:1 (He et al., 2019). While Chl *a*_*PD*_ was shown to be the primary electron acceptor, we demonstrated that the primary electron donor, P_840_, contains a dimer of Zn^2+^-BChl *a*_*P*_′ molecules. The ^14^N and ^67^Zn hyperfine couplings (Table 2) and DFT calculations have indicated that the electron spin density is distributed nearly symmetrically over the two Zn^2+^-(B)Chl *a*_*P*_′ molecules of P_840_^⋅+^ (Charles et al., 2020), as expected in a homodimeric RC. To our knowledge, this is the only example of a photochemical RC in which the (B)Chl molecules of the primary donor are metalated differently from those of the antenna.

### Computational Studies of the Spin Density Distribution

The electronic structure of the oxidized primary donors, P_700_^⋅+^, P_680_^⋅+^, P_865_^⋅+^, and P_960_^⋅+^, of the heterodimeric RC, PS I, PS II and the bRC, have been probed by semi-empirical molecular orbital, quantum mechanics/molecular modeling (QM/MM) and density functional theory (DFT) methods. Early RHF-INDO/SP calculations by Lubitz and coworkers suggested that P_700_^⋅+^ of PS I is formed by a dimer of Chl *a* and Chl *a’* molecules, Chl_1A_ and Chl_1B_, respectively, with an asymmetric charge and electron spin density distribution in favor of the Chl *a* half of the dimer (Käβ et al., 1995; Webber and Lubitz, 2001; Plato et al., 2003). The predicted asymmetry of the charge and spin density distribution across Chl_1A_ and Chl_1B_ was in agreement with previously reported EPR and ENDOR spectroscopy studies (Käβ et al., 2001). Interestingly, the stepwise inclusion of the electrostatic interactions of the Chl_1A_ and Chl_1B_ molecules of P_700_^⋅+^ with the neighboring amino acid residues, such as, Thr743_PsaA_, which forms a putative hydrogen bond with the keto group of Chl *a’*, and His680_PsaA_ and His660_PsaB_, serving as axial ligands to the Mg atoms of Chl_1A_ and Chl_1B_, respectively, led to systematic enhancement of the electronic asymmetry that yielded a spin density ratio of almost 5:1. Molecular orbital calculations indicated that hydrogen bonding specifically stabilized the Chl_1B_ molecule of the dimer, which suggested that the unpaired electron of P_700_^⋅+^ would predominantly reside at this site. This was corroborated by the DFT calculations performed by Sun and coworkers, who found that the asymmetry of the spin density of P_700_^⋅+^ was mainly due to the hydrogen bond to the 13^1^-keto-O group of Chl_1A_ (Sun et al., 2004). More recently, using QM/MM methods Saito and Ishikita (Saito and Ishikita, 2011) estimated the asymmetry of spin density of P_700_^⋅+^ as 22.4:77.6 in favor of the Chl_1B_ molecule. This ratio was in good agreement with the experimental value of 25:75–20:80 that was obtained for the spin density distribution for P_700_^⋅+^ of PS I from spinach (Davis et al., 1993) and 15:85 for P_700_^⋅+^ of PS I from *Thermosynechococcus elongatus* (Käβ et al., 2001). The general consensus has been that there are three factors that significantly contribute to a larger spin population of Chl_1B_ relative to that of Chl_1A_, namely, (i) the presence of the Thr743_PsaA_ residue that forms a hydrogen bond with the 13^1^-keto-O group of Chl_1A_, which is absent in Chl_1B_, (ii) the identity of Chl_1A_ as a Chl *a* epimer, which leads the methyl ester group of Chl_1A_ and Chl_1B_ to be oppositely oriented with respect to the chlorin plane and (iii) the conserved pair of Arg750_PsaA_ and Ser734_PsaB_ residues that interact with Chl_1A_ and Chl_1B_.

The charge and spin density distribution of P_680_^⋅+^ of PS II has also been studied by DFT and QM/MM methods. The charge distribution determined by natural population analysis indicated that the positive charge of P_680_^⋅+^ was significantly delocalized over the two Chl *a* molecules, Chl_1A_ and Chl_1B_, with a slight bias in favor of the Chl_1B_ molecule (ratio of 0.46:0.54) (Takahashi et al., 2008). However, the charge delocalization, and similar spin density distribution, on Chl_1A_ and Chl_1B_ of P_680_^⋅+^ in this study (Takahashi et al., 2008) was in contrast with experimental observations that the charge is mostly localized on one of the Chl *a* molecules, Chl_1A_, of P_680_^⋅+^ (Rigby et al., 1994b). More recently, the delocalization of the charge and spin density across the Chl_1A_ and Chl_1B_ dimer of P_680_^⋅+^ was studied by Saito and coworkers using QM/MM methods (Saito and Ishikita, 2011). The Chl_1A_:Chl_1B_ charge and spin density distribution was found to be 76.9:23.1 and 80.6:19.4, respectively, based on the complete structure of PS II that was obtained from the 1.9 Å X-ray crystal structure (Umena et al., 2011). The calculated spin density distribution was more asymmetric than the charge delocalization in the previous QM/MM study, which was also observed in computational studies of the other oxidized heterodimeric primary donors. The ratio of the spin density distribution on the Chl_1A_ and Chl_1B_ dimer of 80.6:19.4 was in agreement with the ratio of 82:18 that was obtained from experimental ^1^H ENDOR spectroscopy of P_680_^⋅+^ of PS II from spinach (Rigby et al., 1994a) and the ratio of 80:20 from flash-induced spectroscopic studies of PS II from *Synechocystis* sp. PCC 6803 (Diner et al., 2001). This suggested a preferential localization of the cationic state on Chl_1A_ over Chl_1B_ irrespective of the homology of the protein sequences between the D1 and D2 polypeptide subunits of PS II. Interestingly, the removal of the protein subunits of PS II in the QM/MM calculations yielded an isolated Chl_1A_:Chl_1B_ ratio of 57.5:42.5 in vacuum. The significantly lower ratio of Chl_1A_:Chl_1B_ in the absence of the polypeptide subunits, in comparison with that obtained in their presence, suggested that the remarkable asymmetric distribution of the cationic state among Chl_1A_ and Chl_1B_ of P_680_^⋅+^ was not due to the geometry of the Chl molecules but due to the asymmetric protein environment provided by PS II. Based on this observation, it was concluded that the Chl_1A_:Chl_1B_ ratio of ∼ 80:20 was mainly due to the difference in the amino acid residues that were interacting with Chl_1A_ and Chl_1B_ of P_680_.

Initial semi-empirical RHF-INDO/SP calculations of the ^15^N hyperfine couplings of P_865_^⋅+^ of the bRC had yielded a spin density ratio of 1.8:1 across the BChl_1A_ and BChl_1B_ dimer, which appeared to be in agreement with the early ^1^H ENDOR data (Lendzian et al., 1992). However, later ENDOR, ESEEM and HYSCORE spectroscopy measurements of both single-crystal and powder samples of the bRC from *Rba. sphaeroides* and *Rps. viridis* indicated that the ratio of the electron spin density distribution is 3:1 or 5:1 in favor of BChl_1A_ (Lendzian et al., 1988, 1992, 1993). Subsequent semi-empirical molecular orbital calculations by Lubitz and coworkers on the oxidized primary donor, P_865_^⋅+^, using RHF-INDO/SP methods (Käβ et al., 1995) reproduced the experimental isotropic hyperfine couplings to within 10% error, which facilitated the assignment of the hyperfine couplings of all of the nitrogen atoms of the BChl_1A_ and BChl_1B_ dimer and a ratio of the electron spin density distribution of 5:1 was deduced from the measured and assigned hyperfine couplings of the ^15^N atoms of P_865_^⋅+^. Most recently, the asymmetry of the spin density distribution and electronic coupling of the (B)Chl molecules of the oxidized primary donor, P_865_^⋅+^, from *Rba. sphaeroides*, as well as P_700_^⋅+^ and P_680_^⋅+^, of PS I and PS II from *Synechococcus elongatus*, and *Thermosynechococcus vulcanus*, respectively, was estimated by frozen-density embedding diabatic (FDE-diab) methodology (Artiukhin and Neugebauer, 2018). The calculated ratio of spin densities was in agreement with previous experimental results for PS II and the bRC, where 82% and 66% of the spin density of P_680_^⋅+^ and P_865_^⋅+^ was found to be located on the Chl_1A_ molecule.

Density functional theory and QM/MM methods are powerful tools for the investigation of the electronic structure of the oxidized primary donors. As described above, there are several reports in literature on computational studies of P_700_^⋅+^, P_680_^⋅+^, P_865_^⋅+^, and P_960_^⋅+^ of PS I, PS II and the bRC from *Rba. sphaeroides* and *Rps. viridis*, respectively. However, to our best knowledge quantum-mechanical calculations to determine the hyperfine and quadrupolar parameters of the ^14^N atoms of the primary donors of the homodimeric RCs, with the exception of the *Cab*. RC (Charles et al., 2020), are lacking at this time. This may be because the high-resolution structures of homodimeric RCs have been determined only very recently. In order to facilitate a comparison of the oxidized primary donor of hetero- and homodimeric RCs, we performed DFT calculations on computational models of P_800_^⋅+^ and P_840_^⋅+^ of the HbRC and GsbRC from heliobacteria and green sulfur bacteria, respectively. The goal is to obtain a better understanding of the effect of the relative geometry, symmetry and protein matrix effects on the electronic structure of the dimeric BChl molecules in the oxidized primary donor of homodimeric RCs.

In order to assess the computational methods that were employed in this study, we performed DFT calculations on models of P_700_^⋅+^, P_680_^⋅+^ P_865_^⋅+^, and P_960_^⋅+^ that were derived from the X-ray crystal structure of PS I (Jordan et al., 2001) (PDB ID: 1jb0), PS II (Umena et al., 2011) (PDB ID: 3wu2) and the bRC from *Rba. sphaeroides* (PDB ID: 1pcr) (Ermler et al., 1994) and *Rps. viridis* (PDB ID: 1prc) (Deisenhofer et al., 1995). The computational model of each primary donor included the dimeric (B)Chl molecules, (B)Chl_1A_ and (B)Chl_1B_, the axial histidine ligands of the (B)Chls and proximal hydrogen-bonding and hydrophobic residues in the protein matrix as observed in the respective structures (Figures 4C,F, 7C,F). The dimeric models contained the complete (B)Chl molecules with the exception that the phytol tail was truncated by a methyl group after 23 carbon atoms. Please note that there may be an effect of the accessory (B)Chl molecules, (B)Chl_2A_ and (B)Chl_2B_, on the spin density distribution of the oxidized primary donors, which was not investigated in this study.

The single-point energy of each model was calculated employing the hybrid-generalized gradient approximation (hybrid-GGA) B3LYP functional (Dunham et al., 1971; Becke, 1988; Lee et al., 1988) along with the special EPR-optimized EPR-II (Barone, 1995) basis set for the lighter atoms and 6-31G(d) for magnesium, respectively, for most calculations, and the valence polarization basis sets (SVP and TZVP) (Schäfer et al., 1992; Weigend and Ahlrichs, 2005; Weigend, 2006) with the decontracted auxiliary basis sets (i.e., the coulomb fitting def2/J) (Weigend, 2006) when necessary. The calculations were performed in the spin-unrestricted mode for nuclear quadrupole couplings and isotropic hyperfine interactions of pyrrole nitrogen atoms. To account for the influence of solvent effects, a model of uniform dielectric constant of solvents using the conductor-like polarizable continuum model (CPCM) was used in the calculations (Klamt and Schüürmann, 1993; Cossi et al., 2003). The CPCM used a dielectric constant, ε, of 4.0 for incorporating the effects of the protein environment in all of the DFT calculations. All of the calculations included dispersion correction using a DFT-D3 approach with Becke-Johnson damping (D3BJ). In order to estimate the effect of the exchange functional, the hyperfine tensor calculations were also performed with the hybrid-meta-GGA TPSSh (Tao et al., 2003) functional along with the chain of spheres (RIJCOSX) (Staroverov et al., 2003; Neese et al., 2009) approximation and an EPR-II (Barone, 1995) and 6-31G(d) basis set for the lighter atoms and magnesium, that were previously used in the single-point energy calculations. The DFT calculations of P_700_^⋅+^, P_680_^⋅+^, P_865_^⋅+^, and P_960_^⋅+^ were validated by comparison of the calculated and previously published experimental hyperfine coupling constants of the ^14^N atoms. Although the absorbance features of chromophores have been reasonably estimated through quantum mechanical calculations [e.g., the primary donor of *Rba. sphaeroides* (Eccles et al., 1988)], we focused on the prediction of hyperfine coupling constants for the purpose of this review article.

As a starting point for the DFT calculations, we selected a simple computational model for P_700_^⋅+^ that was comprised only of the Chl_1A_ and Chl_1B_ molecules. In this model, the electron spin density across the singly occupied molecular orbital (SOMO) of isolated Chl_1A/1B_^⋅+^ was distributed predominantly on the Chl_1A_ molecule. This was in contrast with experimental findings where the electron spin was predominantly located on Chl_1B_ (see above). To determine the effects of protein-cofactor interactions, we systematically expanded the computational model of P_700_^⋅+^ to include the axial ligands, His680_PsaA_ and His660_PsaB_, putative hydrogen-bonding residues, Thr743_PsaA_, Tyr735_PsaA_, and Tyr603_PsaA_ and the hydrophobic residues, Leu630_PsaB_ and Leu650_PsaA_. As observed in Figure 4C, the protein matrix effects appear to be completely asymmetric across the Chl_1A/1B_ dimer of P_700_ as the hydrogen bonding and electrostatic interactions are limited to Chl_1A_, while the hydrophobic effects are localized in the vicinity of Chl_1B_. We observed that despite the presence of the axial His ligands and putative hydrogen bonding residues, the asymmetric electron spin density distribution in the SOMO that was observed in the DFT calculations was still in favor of Chl_1A_, albeit there was a slight change in the distribution of the spin density within the Chl_1A_ molecule. Interestingly, it was the introduction of the hydrophobic effects from the addition of the residues, Leu630_PsaB_ and Leu650_PsaA_, which resulted in a shift of the spin density distribution in favor of the Chl_1B_ half of the dimer of P_700_^⋅+^.

For the DFT calculations of P_865_^⋅+^ of the bRC from *Rba. sphaeroides*, the computational model included the Tyr210_L_, Val157_M_, His202_L_, Thr186_L_, His168_M_, and His173_M_ residues that are proximal to the BChl_1A_ and BChl_1B_ molecules in the X-ray crystal structure (Figure 7C; Ermler et al., 1994). Similarly, the computational model of P_960_^⋅+^ of the bRC from *Rps. viridis* contained the Val157_M_, Tyr195_L_, His200_L_, Leu184_L_, His168_M_, Trp167_M_, His173_M_, and Thr248_M_ residues (Figure 7F). We observed that the calculated electron spin density distribution in the SOMO of P_865_^⋅+^ and P_960_^⋅+^ was in favor of the BChl_1A_ molecule, both in the absence and presence of the hydrogen-bonding and hydrophobic amino acid residues. We observed a similar trend in the DFT calculation of P_680_^⋅+^ of PS II with a model comprised of the Chl_1A_ and Chl_1B_ dimer with the Ser282_D1_, Leu182_D2_, Met183_D1_, His197_D1_, and His198_D2_ residues (Figure 4F), where the spin density in the SOMO was located on the Chl_1A_ half of the dimer. The localization of the electron spin density on the (B)Chl_1A_ molecule of P_865_^⋅+^, P_960_^⋅+^, and P_680_^⋅+^ was in contrast with that of P_700_^⋅+^, where the asymmetric distribution of the electron spin density was in favor of the Chl_1B_ molecule.

The hyperfine coupling constants of the ^14^N atoms of the oxidized primary donors, (B)Chl_1A_ and (B)Chl_1B_, of P_700_^⋅+^, P_680_^⋅+^, P_865_^⋅+^, and P_960_^⋅+^ that were obtained from the DFT calculations are presented in Table 3. In the case of P_700_^⋅+^ and P_865_^⋅+^ of PS I and the bRC from *Rba. sphaeroides*, respectively, a comparison of the experimental (Table 2) and calculated (Table 3) ^14^N hyperfine couplings indicates that the calculations corroborate the experimental values obtained by ENDOR, ESEEM and HYSCORE spectroscopy. Further, the ratio of the asymmetric spin density distribution across the (B)Chl_1A_ and (B)Chl_1B_ dimer of P_700_^⋅+^ and P_865_^⋅+^ that was estimated from the spin populations was 5.7:1 and 5.6:1, respectively, which was in good agreement with the approximate estimate of Chl_1B_:Chl_1A_:5:1 and BChl_1A_:BChl_1B_:5:1 that was obtained from spectroscopic measurements of P_700_^⋅+^ and P_865_^⋅^+ (Käβ et al., 1995; Käβ et al., 1995; Käβ and Lubitz, 1996; Deligiannakis and Rutherford, 2001; Käβ et al., 2001; Chestnut et al., 2021). To our best knowledge, the ^14^N hyperfine coupling constants of P_680_^⋅+^ are not available in literature. However, the asymmetric spin density distribution that was determined in the DFT calculations of P_680_^⋅+^ of Chl_1A_:Chl_1B_:6:1 was roughly in agreement with the ratio of Chl_1A_:Chl_1B_:80:20 in literature. The broad agreement between the calculated and experimental hyperfine coupling constants and the high asymmetry of the electron spin density distribution of the oxidized heterodimeric primary donors indicates that the DFT methods used in this study are comparable with the experimental data in literature. However, it is important to note that the Kohn-Sham DFT methods employed may have resulted in slight over delocalization of the electron spin density across the oxidized primary donors (Artiukhin and Neugebauer, 2018). Further studies are in progress to address this possibility.

**TABLE 3:** Calculated ^14^N isotropic hyperfine coupling parameters of the nitrogen atoms of P_700_^⋅+^, P_865_^⋅+^, P_960_^⋅+^, P_680_^⋅+^, P_800_^⋅+^, P_840_^⋅+^ (of the GsbRC).

Nitrogen	P_700_^⋅+^ A_iso_ [MHz]	P_865_^⋅+^ A_iso_ [MHz]	P_960_^⋅+^ A_iso_ [MHz]	P_680_^⋅+^ A_iso_ [MHz]	P_800_^⋅+^ A_iso_ [MHz]	P_840_^⋅+^ A_iso_ [MHz]
**N^I^**	2.88	2.88	2.76	3.72	−0.68	1.10
**N^II^**	2.34	2.59	2.26	2.44	−1.01	1.26
**N^III^**	1.64	2.29	2.17	2.19	−0.90	1.37
**N^IV^**	1.26	1.67	1.75	1.55	−1.29	1.61
**N^V^**	0.39	0.35	0.30	0.40	−0.67	1.10
**N^VI^**	0.36	0.15	0.20	0.30	−1.01	1.27
**N^VII^**	0.18	0.14	0.15	0.10	−0.91	1.38
**N^VIII^**	0.11	0.12	0.13	0.10	−1.29	1.63

The DFT calculations on the oxidized heterodimeric primary donors of PS I, PS II and the bRC were consistent the magnetic parameters and asymmetric electron spin distribution over the (B)Chl dimer of P_700_^⋅+^, P_680_^⋅+^, P_865_^⋅+^, and P_960_^⋅+^. Following which, we performed DFT calculations to understand better the effects of the electronic structure and local symmetry on the hyperfine and quadrupolar parameters of the ^14^N atoms in the oxidized primary donors, P_800_^⋅+^ and P_840_^⋅+^, of the RC from the HbRC and GsbRC, respectively. The coordinates for the atoms in the computational models of P_800_ and P_840_ were derived from the structures of the HbRC and GsbRC from *H. modesticaldum* (PDB ID: 5v8k) (Gisriel et al., 2017) and *C. tepidum* (PDB ID: 6m32) (Chen et al., 2020), respectively. Similar to the computational models of the heterodimeric primary donors, the models for the homodimeric ones included the dimeric BChl molecules, BChl_1A_ and BChl_1B_, and the axial ligands to each BChl monomer (Figures 8C,F). Once again, the models contained the complete BChl molecules with the exception that the farnesol tail was truncated by a methyl group after 23 carbon atoms. Both the HbRC and GsbRC are comprised of a homodimeric polypeptide subunit core encoded by the single genes, *pshA* and *pscA*, respectively. However, the symmetry of the homodimeric core of the GsbRC is broken by the presence of an additional PscB polypeptide (Chen et al., 2020). Additionally, while the primary donor, P_840_, of the GsbRC contains a symmetric BChl *a’* dimer with four hydrogen-bonding residues (Figure 8F), two on each BChl molecule, P_800_ of the HbRC is comprised of a dimer of BChl *g*′ molecules that lack hydrogen-bonding interactions (Figure 8C). However, it is expected that the effects of the hydrogen-bonding interactions of P_840_ of the GsbRC will impact the BChl molecules equally, which will not influence the localization of the charge or spin density distribution. Since there are no residues that appear to be participating in hydrogen bonds with P_800_ of the HbRC, the spin density is also expected to be completely symmetric.

**FIGURE 11:**
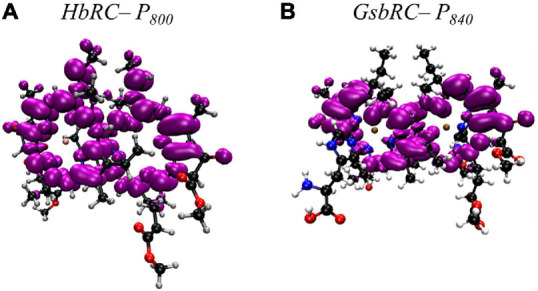
Electron spin density distribution on the Chl_1A_ macrocycles of the homodimeric oxidized primary donor **(A)** P_800_^⋅+^ of the HbRC from *Heliobacterium modesticaldum* (PDB ID: 5v8k) (Gisriel et al., 2017) and **(B)** P_840_^⋅+^ of the GsbRC from *Chlorobaculum tepidum* (PDB ID: 6m32) (Chen et al., 2020) in the singly occupied molecular orbital (SOMO) as determined through DFT calculations.

The DFT calculations of the oxidized primary donor models, P_800_^⋅+^ and P_840_^⋅+^, of the HbRC and GsbRC were also performed with B3LYP level of theory using an EPR-II and 6-31G(d) basis set for the lighter atoms and Mg, respectively. The calculated ^14^N hyperfine coupling constants (Table 3) and the spin populations of BChl_1A_ and BChl_1B_ that were obtained from the DFT calculations indicated that the ratio of the electron spin density distribution across the dimer of P_800_^⋅+^ and P_840_^⋅+^ of the HbRC and GsbRC, respectively, are completely symmetric. The unpaired electron spin density distribution in the singly occupied molecular orbital (SOMO) of P_800_^⋅+^ and P_840_^⋅+^ was also symmetric (Figures 11A,B). The calculated hyperfine couplings of the nitrogen atoms of P_800_^⋅+^ of the HbRC and P_840_^⋅+^ of the GsbRC (Table 3) were in agreement with the experimental couplings obtained from ^14^N ESEEM and HYSCORE spectroscopy measurements of P_840_^⋅+^ from *Chlorobium limicola* and *Cab*. *thermophilum* membranes, respectively (Table 2), respectively. This is indeed interesting as P_800_ of the HbRC is comprised of a BChl *g*’ dimer, while P_840_ of *Cab*. *thermophilum* has been shown to a dimer of Zn^2+^-BChl *a*′ molecules. This suggests that the protein environment surrounding P^⋅+^ in the HbRC and GsbRC is likely similar to that of the *Cab. thermophilum* RC.

Although the symmetry of the homodimeric core in the structure of the GsbRC is broken due to the presence of the additional PscB polypeptide, it appears that this did not impact the magnetic parameters of the BChl dimer of P_840_^⋅+^. This finding is in agreement with the ^1^H ENDOR and HYSCORE spectroscopy studies that demonstrated that the electron distribution of P_840_^⋅+^ of the GsbRC from *Chlorobium limicola* was symmetrical in nature (Rigby et al., 1994b). However, there is the possibility that the small asymmetric effects from the extended protein environment, leading to minor deviations from total symmetry were not observed in the DFT calculations. This would most likely be due to the limited computational model of P_840_^⋅+^ that was adopted in this study.

The spatial arrangements of (B)Chl molecules in the primary donor of PS I, PS II, and the bRC are similar, where there are small differences in the inter-cofactor distance and relative orientation of the (B)Chl molecules. However, the protein matrix effects on the two halves of the (B)Chl dimer are highly asymmetric and this results in the localization of the spin density on a single (B)Chl molecule. We observed that the asymmetry of the spin density distribution is not solely caused by the structural differences of the (B)Chl macrocycles, but by the interaction of the cofactors with the protein environment. In contrast, these asymmetric effects are not present in homodimeric RCs, resulting in nearly equal sharing of spin density between the two BChl molecules (Figures 11A,B). The absence of asymmetric electrostatic and/or hydrophobic effects from the surrounding protein environment leads to a symmetric distribution of the charge and spin density in the homodimeric primary donors, P_800_^⋅+^ and P_840_^⋅+^. This effect is also mirrored in the distance between the macrocycles to ensure that spin density is not localized on one molecule. A direct consequence of the symmetric electronic structure of P_800_ and P_840_ is that it allows for electron transfer through both branches of cofactors with equal efficiency, which is not the case with the heterodimers examined in this study.

## Comparison of the Primary Donors in Hetero and Homodimeric Reaction Centers

Overall, the DFT calculations of the hetero- and homodimeric oxidized primary donors indicated that the presence of the surrounding protein matrix has profound effects on the distribution of the electron spin density across the (B)Chl molecules. Although the spatial arrangement of (B)Chl molecules in the primary donor of PS I, PS II, and the bRC are similar, there are small differences in the inter-cofactor distance and relative orientation of the (B)Chl molecules. As expected, at larger inter-molecular separations the spin density distribution becomes more localized on a single (B)Chl molecule. The increased localization of spin density in the cationic state is caused by the absolute magnitudes of the electronic coupling that decreases exponentially with the inter-molecular separation. It appears that this asymmetry of the spin density distribution is not solely caused by the structural differences of the (B)Chl macrocycles, but by the interaction of the cofactors with the protein environment. This is perhaps most apparent in P_700_, where the protein environment is highly asymmetric with respect to hydrogen bonds and non-polar residues around the two Chls. Interestingly, it is the combination of both hydrogen bonds and non-polar residues that provide the preferential localization on Chl_1B_, since, in the absence of the Leu residues, localization is expected to be completely inverted in favor of Chl_1A_. This observation may merit further detailed investigation. In contrast, these asymmetric effects are not present in homodimeric RCs, resulting in nearly equal sharing of spin density between the two BChl molecules.

The protein environment and its effect on the neutral P_700_ state was recently probed by Mitsuhashi et al. using a combination of computational methods (QM/MM/PCM and time-dependent DFT). They found that this asymmetric environment led to a high coupling for the HOMO of the neutral Chl_1A_/Chl_1B_ pair (85 meV), but a low coupling of the LUMO (15 meV) (Mitsuhashi et al., 2021). They concluded that this caused a preferential (but not exclusive) localization of the excited state on Chl_1A_, resulting the strong preference for A-branch electron transfer. Interestingly, this does not extend to the bRC, where the neutral HOMO and LUMO couplings were found to be high (115 and 134 meV, respectively), implying that the excited state is shared nearly equally among the two BChl molecules. The deciding factor in branch specificity instead occurs at the BChl_2A_ level, in line with previous mutagenesis experiments (Kirmaier et al., 2001). It appears that manipulations to the electronic structure of the primary donor alone are not enough to confer the quantitative branch specificity required in Type II RCs, but are sufficient to lend a slight preference to branch usage, as observed in PS I. This may explain why Type II RCs display less asymmetry of protein matrix effects while attaining high asymmetry in the branch usage.

In addition to affecting the electronic coupling between the two (B)Chl monomers, the protein matrix also influences the dimer as a whole. As highlighted in Figure 3, the monomeric (B)Chls as well as the primary donors exist on a wide distribution of possible redox potentials. From most oxidizing species, P_680_ of PS II with a potential of ∼ 1,200 mV, to the least oxidizing, P_800_ of the HbRC at ∼ 225 mV, the *E*_*m*_ values span a range of nearly 1,000 mV, thus showcasing both the versatility and profound impact that the protein matrix effects can have in altering the electronic properties of (B)Chl molecules. An interesting example is the comparison of P_700_ of PS I to the P_865_ and P_960_ of the bRCs. There are significant differences in redox properties of BChl *a* [*E_m_* = + 640 mV (Fajer et al., 1975)], Chl *a* [*E*_*m*_ = + 800 mV (Fajer et al., 1982; Maggiora et al., 1985)], and Chl *b* [*E_*m*_* = + 940 mV (Kobayashi et al., 2007)], and yet the primary donors of P_700_ (composed of Chl *a*), P_865_ (composed of BChl *a*), and P_865_ (composed of BChl *b*) that have nearly the same redox potential of ∼ 500 mV (Moss et al., 1991; Williams et al., 1992; Nagarajan et al., 1993; Lin et al., 1994; Brettel, 1997; Alric et al., 2004). It should be noted that the redox potential for BChl *b* is unknown, but it is likely more oxidizing than BChl *a* and close to that of Chl *a*. Regardless, through a combination of (B)Chl dimerization and implementation of extensive protein-matrix effects, heterodimeric RCs can modulate the redox potential of the primary donors by at least 300 mV as compared to the respective monomers. Perhaps even more impressive is the remarkably precise control that proteins have to tailor their interactions with chemically dissimilar molecules to achieve a highly similar mid-point potential.

Not only are RCs capable of tuning the mid-point potentials of disparate Chls to similar values, but the reverse is also observed, where proteins are able to modify chemically identical primary donors to achieve vastly different redox potentials. Nowhere is this effect more pronounced than in the comparison of P_680_ and P_700_ of PS II and PS I, respectively. Even though P_680_ and P_700_ are both composed of Chl *a* molecules, their redox potentials vary by over 600 mV, with the *E*_*m*_ of P_680_ and P_700_ ∼ 1,200 mV (Rappaport et al., 2002; Ishikita et al., 2005) and ∼ 500 mV, respectively. Indeed, nature is able to take a modestly oxidizing species and form one of the most oxidizing cofactors in biology. Please note that the value of the redox potential of P_700_ is approximate, as it has been shown to vary in different organisms, with P_700_ of spinach PS I reporting a value of +470 and ∼ +400 mV for the cyanobacterium *Gloeobacter violaceus* (Nakamura et al., 2011). Regardless, the difference of ∼700 mV for a dimer of chemically identical Chl molecules is noteworthy. While this difference is staggering, it must be acknowledged that these RCs ultimately are primed to serve vastly different functions. PS I must generate a low potential reductant sufficient to reduce a soluble ferredoxin, while PS II needs to generate a strong oxidant for water splitting at the Mn_4_Ca-oxo cluster.

In contrast to heterodimers, the primary donors of homodimeric reaction centers exhibit remarkably similar midpoint potentials. Redox titrations on the primary donor, P_840_, of the GsbRC yielded a reduction potential of +217 (Azai et al., 2016)–+240 mV (Fowler et al., 1971; Prince and Olson, 1976; Hauska et al., 2001). Related experiments on P_800_ from the HbRC yielded a surprisingly similar *E*_*m*_ of +225 mV (Prince et al., 1985). This is notable given the differences in the surrounding protein environment and chemical identity of the BChl molecules. While they share the same axial ligands, P_800_ lacks any hydrogen bonds while P_840_ has two putative hydrogen bonds to each BChl in the dimer and two unique aromatic residues in close proximity. There are currently no empirical measurements in the literature for the *E*_*m*_ of BChl *g*, so it is difficult to assess the degree to which the redox potential of P_800_ must be altered to achieve a value of +225 mV. However, the fact that the HbRC, containing BChl *g*, can achieve the same redox potential as GsbRC, containing BChl *a*, without the extensive protein-matrix effects suggests a more oxidizing potential of BChl *g*. The reason for the *E*_*m*_ of each RC to shift by ∼ 250 mV in comparison to the heterodimeric Type I RC, PS I, is not well understood, as both RCs are capable of reducing soluble ferredoxins (Seo et al., 2001; Romberger and Golbeck, 2012). It should be noted that the more simplified RCs have fewer electron transfer cofactors and therefore do not need to account for the loss of ΔG. However, that the GsbRC is associated with a cytochrome *c*_551_ that is only 53 mV more negative than P_840_ (Kusumoto et al., 1999) may be a contributing factor. Further insight can be gained from the HbRC, as the BChl *g* pigments that compose the primary donor are subject to relatively controlled oxidation to Chl *a*_ox_ in the presence of light and O_2_. Interestingly, partial oxidation of the pair, resulting in BChl *g*/Chl *a*_ox_ remains functionally active and able to generate a charge-separated state (Ferlez et al., 2015). However, full oxidation of both BChl *g* molecules results in a loss of activity. Whether this loss of activity arises from a decoupling of the dimer, a shifting of its redox potential, or another substantial change to its electronic properties remains an open question.

Alterations to the redox and electronic properties of primary donors and its effects on the efficiency of charge separation have been studied in literature. Interestingly, small changes to the local protein environment can substantially impact the redox potential of the primary donor. Allen and Williams (Allen and Williams, 1995) investigated a series of site-specific genetic variants that either altered, added, or deleted hydrogen bonds to P_865_. Removing all of the hydrogen bonds by replacing a His residue by a Phe decreased the redox potential of P_865_ by 95 mV, while the addition of three His residues resulting in a total of four hydrogen bonds increased the potential by 260 mV. Concomitant with increase of the redox potential was a decrease of the recombination rate of the P_865_^⋅+^Q_A_^⋅–^ state by 60% and an increase in the lifetime of the excited state decay by ∼ 15 times. Similar genetic variants of other bRCs have displayed similar effects (Jia et al., 1993; Wachtveitl et al., 1993a,b). For example, a change in the electrostatic environment when the Tyr210_L_ residue in the bRC from *Rps. viridis* was replaced by Phe, Ile, or Trp, each resulted in a redox potential that was sequentially more positive (Alden et al., 1996). A better understanding of the factors that influence the redox properties of primary donors has allowed for exciting progress in re-directing electron transfer within the bRCs (Wakeham and Jones, 2005; Williams and Allen, 2009).

Nature has many robust tools for the tuning and control of photosynthetic electron transfer cofactors that are tailored to its various needs, especially the primary donors of photosynthetic RCs. It has demonstrated the ability to not only align the redox potential of differing cofactors, but also push identical molecules to extreme oxidative potentials. Protein-matrix effects have a profound impact on the electronic properties of both the neutral and oxidized states of the primary donor, which we are just beginning to understand. As shown in this review, modern computational methods can be employed to model these states and accurately re-create the electronic properties that are observed experimentally. As we are able to understand better the influence of hydrogen bonds, charged and/or hydrophobic residues, the geometry and relative orientation of the (B)Chls, and protein motion (Gorka et al., 2020) on the highly efficient processes of light harvesting and electron transfer, it delivers insight on the evolutionary photosynthetic origins in our past and provides a blueprint for the design of artificial photosynthetic systems in the future.

## Author Contributions

MG, KVL, and JHG were responsible for the writing of the manuscript. MG and KVL were responsible for the geometric analysis of reaction centers, while AB, AM, EG, and KVL performed the computational analysis. All authors contributed to the article and approved the submitted version.

## Conflict of Interest

The authors declare that the research was conducted in the absence of any commercial or financial relationships that could be construed as a potential conflict of interest.

## Publisher’s Note

All claims expressed in this article are solely those of the authors and do not necessarily represent those of their affiliated organizations, or those of the publisher, the editors and the reviewers. Any product that may be evaluated in this article, or claim that may be made by its manufacturer, is not guaranteed or endorsed by the publisher.
